# A tale of two apps: preliminary usability testing of smoking cessation mobile applications among individuals with low socioeconomic status who smoke cigarettes

**DOI:** 10.1186/s12911-026-03393-5

**Published:** 2026-02-27

**Authors:** Michael Wakeman, Gabrielle Zuckerman, Gunnar Baskin, Timothy Gregory, Greg Gruse, Erin Leahy, Brandon Kendrick, Sherine El-Toukhy

**Affiliations:** 1 Division of Intramural Research, National Institute on Minority Health and Health Disparities, National Institutes of Health, 11545 Rockville Pike, Rockville, MD 20852 USA; 2 ICF, Reston, VA USA

**Keywords:** Smoking cessation interventions, Usability testing, Young adults, Mobile applications, User experience

## Abstract

**Background:**

To examine the usability of two smoking cessation apps, Quit Journey, a newly developed app, and QuitGuide, a benchmark app, among young adults who smoked and were socioeconomically disadvantaged, as a primary target population for smoking cessation efforts.

**Methods:**

Ten current cigarette smokers aged 18–29 years who were neither four-year college graduates nor enrollees participated in virtual think-aloud 1-hour usability sessions. Sessions were held on GoTo Meeting, audio- and screen-recorded, and transcribed verbatim. Participants completed tasks on simulated versions of each app. We assessed task completion success rates, time-to-completion in seconds, and post-testing usability measures (e.g., System Usability Scale [SUS]). We used deductive thematic analysis to code qualitative participant feedback for constructs from the Second Unified Theory of Acceptance and Use of Technology and sentiment.

**Results:**

For Quit Journey, task completion rates averaged 55.17% and time-to-completion ranged from 7.00 to 69.00 s. For QuitGuide, completion rates averaged 80.64% and time-to-completion ranged from 4.00 to 54.00 s. SUS scores averaged 69.00 and 81.87 for Quit Journey and QuitGuide, respectively. Participants’ feedback focused mainly on perceived effort and usefulness of apps’ features.

**Conclusions:**

Usability testing revealed deviations from usability guidelines (e.g., confirming user-input), resulting in lower task completion rates and longer completion times, especially for Quit Journey. Resolving usability issues identified in this testing round will help improve user experiences in future iterations of Quit Journey. Iterative usability testing is critical to optimize the effectiveness, efficiency, and user satisfaction of smoking cessation apps and should be integral to their development.

**Supplementary Information:**

The online version contains supplementary material available at 10.1186/s12911-026-03393-5.

## Background

Mobile applications can deliver convenient and accessible smoking cessation interventions that circumvent barriers that often leave traditional cessation support (e.g., medications, counseling) underutilized [1, 2]. This is particularly important for socioeconomically disadvantaged populations that smoke at higher rates than the national average of 11.5% and bear a high burden of smoking-related adverse health outcomes [1]. In 2021, the highest smoking prevalence by educational attainment and income brackets was documented among people with General Education Development (GED), with no high school diploma, and with low income-to-poverty ratio (30.7%, 20.1%, and 18.3%, respectively) [3]. Despite their underuse of traditional cessation support that is attributed to several barriers (e.g., lack of health insurance), [1] people with low socioeconomic status (SES) express interest in self-help technology-based smoking cessation support such as mobile applications [4, 5]. Mobile applications can provide self-help cessation support, either standalone or complementary to other cessation support [1]. To ensure uptake and continuous use of smoking cessation applications, which can potentially improve cessation, [6, 7] developers must adhere to fundamental usability guidelines during app development and evaluate the app’s usability among target audiences.

Usability refers to users’ satisfaction, effectiveness, and efficiency in performing tasks within an app [8, 9]. It is a crucial, yet often overlooked, aspect of the development of mobile health interventions [10]. Usability guidelines pertain to an app’s layout, navigation, visual design, content, and performance, among other components [10–12]. Examples of such guidelines include maintaining simple, consistent, intuitive, and uniform design; using clear-choice navigation menus, identifiable icons, and meaningful headings; making important features accessible from the homepage while affording users the ability to navigate back and/or to the homepage; and providing users adequate feedback [10–12]. Health applications that adhere to these guidelines are satisfying for users, [10] whereas untested apps can lead to frustrating user experiences, underuse or non-sustained app use, and less effective interventions [13].

Usability testing determines users’ experiences utilizing an app, which is commonly assessed by their satisfaction with the app and their ability to complete specified tasks successfully and efficiently [8]. This testing can help developers identify unforeseen issues encountered by app users before its dissemination. For instance, user testing of smoking cessation mobile apps among women identified accessibility (e.g., compatibility with different phone models) and design (e.g., unclear icons) issues [14]. Usability testing is particularly important as target users evaluate mobile health apps differently than developers as evidenced by discrepancies in expert ratings and user testing [15].

Although usability is a critical element in the development of effective mobile health interventions, [13] usability testing is seldom conducted [16]. There is limited research supporting commercially available smoking cessation apps, whereas only a handful of usability studies are available for research-backed smoking mobile apps [16]. There are scarce examples of usability testing among specific (e.g., individuals who are pregnant and smoke) or general populations that smoke [14, 16–19]. To our knowledge no usability testing of any smoking cessation app has been conducted among people with low SES who smoke [16]. This raises concerns about the usability of existing smoking cessation apps for disadvantaged populations who have unique characteristics that should be accommodated if they are to fully benefit from smoking cessation mobile interventions [20]. Indeed, there is evidence that some of the most popular mobile health apps are less likely to be usable by disadvantaged populations [21].

In this study, we aimed to compare the usability of two smoking cessation mobile applications, Quit Journey, a newly developed app, and QuitGuide, a benchmark app. Quit Journey is a multi-component smoking cessation mobile intervention with features rooted in behavioral change techniques [22]. Briefly, Quit Journey includes foundational features common to smoking cessation apps (e.g., providing cessation educational information). The app includes a carbon monoxide monitoring feature, an attentional bias modification mobile game, and digital motivational incentives. The app also includes a library of on-demand and just-in-time support messages. User feedback on the features of Quit Journey is available elsewhere [23–26]. QuitGuide is a publicly available, free-of-charge smoking cessation app operated by the National Cancer Institute as part of its Smokefree.gov Initiative [27]. QuitGuide has 195,476 unique downloads since its release in 2010. This is the first usability testing of Quit Journey. Conversely, previous usability testing of QuitGuide with people with mental illness who smoke showed high task completion rates but below-average System Usability Scale (SUS) scores [18, 19]. This study satisfies the preparation phase of the multiphase optimization strategy (MOST) [28] for developing and evaluating smoking cessation interventions by helping us identify usability issues related to the information structure, design and graphics, and content delivery in Quit Journey against QuitGuide as a benchmark app. Usability issues identified in this stage can be resolved in future iterations of the Quit Journey to achieve high user satisfaction, effectiveness, and efficiency in task performance, particularly among people with low SES who smoke.

## Methods

### Participants and recruitment

Userworks, Inc. (Silver Spring, MD) recruited a convenience sample of 10 participants using its research panels and commercial platforms (e.g., Craigslist) between March and April 2020. Candidates were contacted via email and those interested were screened for eligibility over the phone. Eligible participants were 18–29 years old given reduced mortality risk associated with quitting at younger ages, especially by age 30; [29–31] neither four-year college graduates nor enrolled in a four-year college, as an indicator of low SES; [32–34] current cigarette smokers who smoked at least 100 cigarettes in their lifetime and smoked every day or some days; willing to quit within 6 months; not using any smoking cessation aids or noncigarette combustible tobacco products (e.g., cigars); smartphone owners; and English speaking. Participants received $100 in compensation.

### Procedures

We conducted 10 usability sessions using think-aloud protocols, which require participants to verbalize their thoughts as they complete predefined usability tasks [35]. Five participants evaluated the usability of each app. Prior studies show that data from 5 participants reveal up to 85% of the usability problems of the system being evaluated [36]. We opted to have participants test one app to ensure that their feedback reflected experiences with a single app, rather than comparisons of the two, especially given some feature similarities between the two apps (e.g., mood recording).

First, participants freely explored the app for 5 min. Then, they completed 6 tasks for Quit Journey (e.g., redeem a digital coupon) and 8 tasks for QuitGuide (e.g., record their mood) that involved the primary features of each app. Following each task, participants answered questions on the feature’s perceived usefulness and ease of use informed by Second Unified Theory of Acceptance and Use of Technology (UTAUT2) [37]. Finally, participants answered a post-test questionnaire that included a 10-item SUS, ranging from 0 to 100 with 68 being an acceptable usability benchmark, [38] and 5-point Likert-scale items on intentions to use the app, mobile application loyalty, and app star rating [39, 40]. Participants also answered one question on the intended number of app uses over the next two months and another on their willingness to pay for the app (See Supplementary note 1 for a complete list of usability tasks and post-test questions).

The one-hour usability testing sessions were conducted on GoTo Meeting using Axure RP and AxShare. Axure RP is a wireframing tool and Axshare is the accompanying website that hosts wireframes to make them clickable. Axure RP and Axshare simulate the mobile app on a participant’s device (e.g., computer, smartphone), which allows participants to click through the simulated mobile app to explore it and complete tasks. We audio recorded the sessions and video recorded participants’ screens. Sessions were transcribed verbatim using GoTo Meeting’s built-in transcription feature and later verified by 3 members of our staff. All participants verbally consented to participate before recordings began.

### Data analysis

Two coders, MW and GZ, independently assessed completion success/failure and time-to-completion for all tasks. We marked a task as successfully completed if participants accomplished it without guidance, arrived at the correct app location, or described how they would accomplish it in case of unclickable links or icons in the simulated version of each app (list available upon request). For task completion times, we recorded the time from when the moderator had finished instructing the participant on a task and answering any clarifying questions to when the participant stated/asked if they had completed the task or asked for guidance. Both coders agreed 100% on task completion success/failure determination and the average difference between coders for time-to-completion was < 3 s.

We followed a deductive thematic approach to analyze the qualitative data [41]. Using ATLAS.ti software (version 8, ATLAS.ti Scientific Software Development GmbH), MW coded participants’ feedback for salient themes based on five UTAUT2 constructs: effort expectancy, facilitating conditions, hedonic motivation, performance expectancy, and social influence [37]. Quotes that did not fit under any of these constructs were coded as “not applicable.” Additionally, transcripts were coded for sentiment (i.e., negative, neutral, positive), app task, general feedback mentioned outside predefined tasks, intent/willingness to use the feature or app, and user suggestions. Theme definitions appear in Supplementary Table 1 [23–26, 42, 43]. Quotes could be coded with multiple semantic domains, but only one theme could be selected from each domain. SEL reviewed the coding, and disagreements were resolved through discussion.

## Results

Participant characteristics appear in Table 1. The sample was 30% female, 80% White, 30% high school graduates, and 80% daily smokers. Below we present data on task completion rates and time supported by quotes from participants.

**Table 1:** Participant characteristics

App	ID	Sex	Race and/or ethnicity	Highest level of education	Smoking frequency	Quit timeframe	Smartphone operating system
QuitGuide	P01	Male	White	HS graduate	Every day	7 days	iPhone
	P02	Male	White	Some college, ND	Every day	30 days	Android
	P03	Male	White	HS equivalent	Every day	7 days	Android
	P04	Male	White	Some college, ND	Every day	30 days	iPhone
	P05	Female	AIAN	Some college, ND	Some days	7 days	iPhone
Quit Journey	P06	Female	White	Some college, ND	Every day	6 months	iPhone
	P07	Male	White	Some college, ND	Some days	7 days	iPhone
	P08	Male	White	Some college, ND	Every day	6 months	Android
	P09	Female	Black/AA	HS equivalent	Every day	30 days	Android
	P10	Male	White	Some college, ND	Every day	30 days	iPhone

ID= Identification number, AA= African American, AIAN= American Indian/Alaska Native, HS = High school, ND = No degree

### Task completion rate and time

Task completion rates, average task completion time, and average task duration time for Quit Journey and QuitGuide appear in Table 2 and participant-level results appear in Supplementary Table 2. The average task completion success rate was 55.17% for Quit Journey. Tasks with the lowest completion success rates (20.00%) were requesting location-based support and liking messages in the app’s message library. Mood recording was the only task with a 100.00% completion success rate, while the remaining three tasks (i.e., going through carbon monoxide monitoring steps, playing the Fruit Squish game, redeeming a coupon) had completion rates ≥ 60%. Apart from carbon monoxide monitoring, participants completed all tasks in < 1 min. Average task completion time for successfully completed tasks ranged from 7.00 s for accessing support messages to 69.00 s for carbon monoxide monitoring, whereas average task duration time regardless of task success/failure determination ranged from 37.20 s for playing Fruit Squish to 85.20 s for accessing support messages.

**Table 2:** Task completion rates and times for Quit Journey and QuitGuide

App	Task no.	Task description	Task completion success Num/den (%)	Task completion time^*^ M (SD) in seconds	Task duration^**^ M (SD) in seconds
Quit Journey	1	Record your current mood	4/4 (100.00)	50.75 (35.68)	50.75 (35.68)
	2	Program the app to help you whenever you are at your current location by sending support messages	1/5 (20.00)	38.00 (0)	63.40 (44.81)
	3	Say the steps you would take to use the smokerlyzer device to measure the level of carbon monoxide in your breath	3/5 (60.00)	69.00 (31.02)	70.60 (29.91)
	4	Access the message library on the app and pull two or three messages you like	1/5 (20.00)	7.00 (0)	85.20 (86.52)
	5	Find the game Fruit Squish and play it briefly	4/5 (80.00)	30.00 (9.40)	37.20 (16.67)
	6	Redeem a coupon of your choice	3/5 (60.00)	23.33 (17.61)	56.20 (49.30)
QuitGuide^***^	1	Find out whether this app contains information about quitting smoking	4/4 (100.00)	16.25 (6.86)	16.25 (6.86)
	2	Set up a quit date a week from now	4/4 (100.00)	32.00 (5.24)	32.00 (5.24)
	3	Contact the quitline on this app	0/4 (0)	-	69.50 (24.45)
	4	Record your current level of craving for a cigarette	4/4 (100.00)	24.00 (8.86)	24.00 (8.86)
	5	Find information on distractions from cravings in this app	3/4 (75.00)	35.00 (14.44)	38.50 (13.90)
	6	Submit a personal reason for quitting	3/4 (75.00)	20.00 (25.46)	22.00 (22.32)
	7	Program the app to help you at a specific location by sending support messages when you are nearby	4/4 (100.00)	54.00 (24.25)	54.00 (24.25)
	8	Stored in the app is the number of days a previous user was smoke-free, and the minutes and dollars the user saved by being smoke-free. Find these numbers.	3/3 (100.00)	4.00 (2.16)	4.00 (2.16)

Num = Numerator, den = Denominator, M = Mean, SD = Standard deviation

Denominators do not total 5 because of lost recordings or skipped tasks (e.g., for time restraints)

^*^Averaged over the total number of people who completed the task as shown in the numerator of the task completion success column

^**^Averaged over the total number of people who undertook the task (regardless of success/failure determination) as shown in the denominator of the task completion success column

^***^Session recording was lost for P05

The average task completion success rate was 80.64% for QuitGuide, with five tasks having perfect completion rates (i.e., finding cessation information, setting a quit date, recording cravings, requesting location-based support, viewing user cessation-related statistics). Average task completion times for successfully completed tasks were all < 1 min and ranged from 4.00 s for viewing user statistics (e.g., days smoke-free) to 54.00 s for requesting location-based support. Average task duration time regardless of task success/failure determination ranged from 4.00 s for viewing user statistics to 69.50 s for contacting a quitline.

### Qualitative feedback on usability tasks

Most of the quotes focused on ease of use and perceived usefulness of each app (Table 3). Other technology acceptance factors (e.g., facilitating conditions, hedonic motivation) were discussed to a lesser extent. We show sample quotes of issues that participants highlighted with participant attribution (e.g., P01) and the referenced app (i.e., QJ for Quit Journey, QG for QuitGuide). A compilation of all extracted quotes, including feedback on task performance, general app feedback, and suggestions to improve both apps, appear in Supplementary Tables 3–5.

**Table 3:** Distribution of number of quotes by technology acceptance constructs and sentiment for Quit Journey and QuitGuide

Technology acceptance constructs	Quit Journey				QuitGuide			
	Sentiment			Total *n* (%)	Sentiment			Total *n* (%)
	Negative *n* (%)	Neutral *n* (%)	Positive *n* (%)		Negative *n* (%)	Neutral *n* (%)	Positive *n* (%)	
Effort Expectancy	35 (43.20)	9 (11.11)	37 (45.67)	81 (39.13)	11 (19.64)	4 (7.14)	41 (73.21)	56 (31.81)
Facilitating Conditions	7 (31.81)	9 (40.90)	6 (27.27)	22 (10.62)	7 (38.88)	5 (27.77)	6 (33.33)	18 (10.22)
Hedonic Motivation	4 (66.66)	1 (16.66)	1 (16.66)	6 (2.89)	0 (0)	0 (0)	0 (0)	0 (0)
Performance Expectancy	17 (34.00)	4 (8.00)	29 (58.00)	50 (24.15)	1 (1.66)	5 (8.33)	54 (90.00)	60 (34.09)
Social Influence	0 (0)	0 (0)	0 (0)	0 (0)	0 (0)	0 (0)	0 (0)	0 (0)
Not Applicable	11 (22.91)	13 (27.08)	24 (50.00)	48 (23.18)	9 (21.42)	9 (21.42)	24 (57.14)	42 (23.86)
Total	74 (35.74)	36 (17.39)	97 (46.85)	207 (100.00)	28 (15.90)	23 (13.06)	125 (71.02)	176 (100.00)

Column totals add to 100% within each theme, whereas overall row totals add to 100% across a semantic domain

Qualitative feedback from participants highlighted several usability problems. First, ambiguous feature locations, having to click through multiple pages to reach a page, and the lack of navigation between pages emerged as usability problems.

*[What was] difficult [about finding information on distractions] was … you kinda gotta look within a menu within a menu.* (P03, QG)

*There’s no … back button anywhere*,* so I’d have to go straight all the way back to [the] home [page] to get anywhere else.* (P07, QJ)

Second, unintuitive labeling and icons were another usability issue. For example, some participants were confused over the time- and location-based features labeled “help me next time I’m here” and “help me next time tomorrow” in the app.

*There were two things underneath [the mood tracking] saying … help me next time [I’m here and tomorrow]. I’m not really sure why I’d need help doing that.* (P07, QJ)

Third, participants highlighted difficulties with information display such as the need to scroll through content.

*Now [the messages are] going sideways*,* I guess because I’m so used to scrolling up and down*,* sideways … I would have to get used to not skipping too fast.* (P09, QJ)

*When we click on [the cessation information]*,* we have to scroll through the whole thing*,* but if we had like a bulleted list and then subcategories you could click that link and go straight to that … section.* (P04, QG)

Finally, participants stated that the app did not provide confirmation following user inputs, which created ambiguity among participants regarding the success of user input or task status.

*[What was difficult about using the coupons] not knowing if I’m getting my money*,* for one*,* and then I don’t know if it approved me.* (P10, QJ)

Unrelated to usability, technical issues related to non-functional links in the simulated apps and unclear wording of some tasks contributed to failure to complete some tasks and/or prolonged task completion time.

*I feel like I could accomplish [setting a quit date] but … I couldn’t get to the right month … It would be incredibly easy for me to*,* if it wasn’t for that … minor bug … It wasn’t even a second thought of where to go … I remember looking at … my quit plan on the menu*,* and … it’s the second option*,* it’s right there.* (P01, QG)

*Not exactly sure how to … pull these [messages]*,* or I think you could just … like them but I’m not exactly sure.* (P08, QJ)

### Post-testing quantitative usability measures

Descriptive data on post-testing usability measures appear in Table 4. Mean SUS scores for Quit Journey and QuitGuide were 69.00 ± 9.16 and 81.87 ± 6.46, respectively. On average, Quit Journey scored 3.60 on intentions to use the app and 3.80 on intentions to continue to use the app. Quit Journey scored a 4.40 on whether participants would recommend it to others and on whether it would be their first choice for smoking cessation apps. Three participants stated they would use it > 50 times over a two-month period. Two participants (40%) were willing to pay for the app. Quit Journey had a 3.40 average star rating.

**Table 4:** Post-test usability measures

Participant ID	SUS	Intent to use^1^	Continue to use^1^	Recommend app^1^	First choice of app^1^	App use over 2 months^2^	Willing to pay for app	Star rating^3^
**QuitGuide**								
P01^*^	-	-	-	-	-	> 50 times	Yes	4
P02	80.00	3	3	4	2	> 50 times	No	4
P03	92.50	4	4	4	5	10–50 times	Yes	4
P04	75.00	2	2	5	2	10–50 times	No	3
P05	80.00	5	5	5	3	> 50 times	Yes	4
Overall Mean (SD)	81.87 (6.46)	3.50 (1.11)	3.50 (1.11)	4.50 (0.50)	3.0 (1.22)	-	60%	3.80 (0.40)
**Quit Journey**								
P06	77.50	5	5	5	5	> 50 times	Yes	5
P07	52.50	2	2	2	2	0 times	No	1
P08	67.50	4	4	5	5	> 50 times	No	4
P09	77.50	5	5	5	5	> 50 times	No	4
P10	70.00	2	3	5	5	3–10 times	Yes	3
Overall Mean (SD)	69.00 (9.16)	3.60 (1.35)	3.80 (1.16)	4.40 (1.20)	4.40 (1.20)	-	40%	3.40 (1.36)

ID = Identification number, SUS= System usability scale, SD = Standard deviation

^1^ Scale: 1 = Strongly Disagree, 2 = Disagree, 3 = Neutral, 4 = Agree, 5 = Strongly Agree

^2^ Indicates how many times participant would use the app in the next 2 months if they were to quit smoking

^3^ Scale: 1 = One of the worst apps I’ve used, 3 = Average, 5 = One of the best apps I’ve used

^*^ Did not complete post-test questionnaire due to time constraints

QuitGuide scored 3.50 for use and continue to use intentions, 4.50 on whether participants would recommend the app to others, and 3.0 on whether QuitGuide would be their first choice for a smoking cessation app. Three participants stated that they would use the app > 50 times over a two-month period. Three participants (60%) were willing to pay for the app, which had an average star rating of 3.80.

## Discussion

This study reported on the usability of two smoking cessation mobile apps, Quit Journey, a newly developed smoking cessation app, and QuitGuide, a benchmark app. Compared to QuitGuide, Quit Journey underperformed on task completion rates and times, but had an acceptable SUS score and was comparable to QuitGuide on other quantitative usability measures. Failure to complete tasks in Quit Journey, prolonged task completion times, and participants’ qualitative feedback highlighted deviations from usability guidelines including the need for concise labeling, logical pathways to access content, and app feedback on user input. Identifying usability issues in Quit Journey is the first step to resolving them in future iterations of the app [10], which can improve users’ experience and promote its uptake and sustained use. The disproportionate number of available smoking cessation apps in relation to published usability studies indicates that their development is largely uninformed by usability evidence. This study adds to the limited smoking cessation mobile app usability research conducted, especially among disadvantaged and diverse populations [16, 21]. 

Usability testing is often overlooked despite being an important aspect of mobile health apps’ development and an underlying factor in engagement with mobile health interventions, which is associated with improved outcomes [6, 16]. Indeed, prolonged use of smoking cessation apps has been shown to significantly improve cessation. For example, those who used iCanQuit smoking cessation app for either 4 or 26 weeks had 50% and 397% higher odds of cessation at 12-month follow-up compared to those who used the app for one week [6]. However, users of smoking cessation apps do so for only one week [6]. Technical difficulties have been identified as a barrier to user engagement and retention [44] and deviations from usability principles often result in platforms that are difficult to learn, prone to user errors, and less effective [13]. Thus, usability testing is important, particularly for disadvantaged populations who have unique user profiles and needs (e.g., low health or digital literacy) that app developers should accommodate to make health apps engaging and usable by their end users [20, 45]. Popular mobile health apps have had poor usability among under-resourced groups, and barriers such as low confidence and frustration using digital technologies have emerged.[21] Although we did not uncover any issues unique to individuals with low SES, additional studies are needed with other populations who smoke (e.g., older individuals who smoke). 

Failure to complete tasks, prolonged completion times, and user feedback identified here reflect deviations from established usability principles [10]. Consistent with usability recommendations and our qualitative evaluation of both apps, our results showed issues related to feature locations; icon characteristics; menu and screen structure; and content labeling, input, and output. Other issues such as the importance of using accessible language (e.g., clarifying scientific terms such as ‘parts per million’ when showing carbon monoxide readings) and affording users the ability to customize the app (e.g., setting message notification preferences) were discussed to a lesser degree. Notably, two usability tasks were present in both Quit Journey and QuitGuide (i.e., recording mood and craving, requesting location-based support). All participants using QuitGuide successfully completed both tasks, whereas only 20% of participants using Quit Journey were able to successfully request location-based support. This speaks to the importance of repeated usability testing of newly developed apps among their target users [46].

Our results will help us resolve all usability problems in Quit Journey identified in this testing round. We will modify the app to make features accessible through multiple and logical pathways (e.g., the homepage and main menu) within one or two clicks at most. For example, we intend to make Fruit Squish, a smoking cues attentional bias modification game, accessible from the app’s menu instead of only from the cravings tracking page. We intend to rename some icons (e.g., alarm clock for the “I slipped” function) and use concise language for the app’s features (e.g., location- and time-based support). Finally, we intend to include a back button to allow users to navigate between app pages. Given the importance of continuous usability testing of mobile health applications [46], we will conduct additional usability field testing of Quit Journey in a planned optimization study with a large sample of adults who smoke and are with low socioeconomic status where more issues are bound to emerge [47]. This additional testing will complement the results reported here and ensure all modifications made to the app based on the results reported here are acceptable to our target audience.

This study is, to our knowledge, one of the first to test the usability of smoking cessation apps among people with low SES. We employed objective and subjective usability metrics including task completion success/failure, task completion time, and qualitative feedback from participants. Several limitations are noteworthy. The small sample size, while appropriate for this preliminary usability testing phase, is a limitation that will be expanded in a forthcoming field-based usability evaluation of Quit Guide. With the remote setting of our usability tests, participants navigated simulated apps on their computer or phone screens rather than in-person and experienced technical complications that could have negatively affected task completion, times, and overall ratings of both apps. Screen recording was interrupted for one participant and screen and audio recording was lost for another, which resulted in data loss. We used education as an indicator of SES rather than a composite of indictors (e.g., education and income) [32–34]. Furthermore, participants were primarily White, under the age of 30, and likely digitally literate [48]. Accordingly, our results may not be representative of all people who smoke, particularly older individuals and those who are not digitally literate [48, 49].

## Conclusions

This study is one of the first to evaluate the usability of two smoking cessation mobile apps among people with low SES who smoke. Usability testing highlighted problems related to app navigation, design, and content delivery. Resolution of these usability problems will improve user experience and satisfaction of Quit Journey, potentially improving its use and efficacy. This is particularly important for under-resourced groups that could benefit from self-help smoking cessation resources. Recommendations of mobile apps for smoking cessation support should be contingent on appropriate usability testing with target populations, especially those disproportionally affected by smoking, as part of app development and evaluation processes.

## Supplementary Information

Below is the link to the electronic supplementary material.

Supplementary Material 1

## Data Availability

Data is provided within the manuscript or supplementary information files.

## Declarations

### Disclaimer

This research was supported [in part] by the Intramural Research Program of the National Institutes of Health (NIH). The contributions of the NIH author(s) are considered Works of the United States Government. The findings and conclusions presented in this paper are those of the author(s) and do not necessarily reflect the views of the NIH or the U.S. Department of Health and Human Services.

## Abbreviations

SES: Socioeconomic status
SUS: System Usability Scale
UTAUT2: Second Unified Theory of Acceptance and Use of Technology
