# Supplementary material for: A tale of two apps: preliminary usability testing of smoking cessation mobile applications among individuals with low socioeconomic status who smoke cigarettes
Source: BMC Med Inform Decis Mak. 2026 Feb 27;26:105. doi: 10.1186/s12911-026-03393-5 (PMC13049979; doi:10.1186/s12911-026-03393-5)
Supplement: Supplementary file 1 — Supplementary Material 1 [file 12911_2026_3393_MOESM1_ESM.docx]

**Supplementary note 1. Usability test guide and post-test questions.**

We’ve asked you to come here today because we’re testing a mobile app for smoking cessation that we are working on, so we can see what it’s like for actual people to use it.

I want to make it clear right away that we are not testing how well you can use the app. We’re testing the app to understand how easy or difficult it is to use. You can’t do anything wrong here. In fact, this is probably the one place today where you don’t have to worry about making mistakes.

We didn’t make this app, so you aren’t hurting my feelings if you criticize it. My job is to find out what’s wrong with it or where it can be improved. If you think the app is confusing or difficult to use, we need to know honestly what you think so that we can inform makers of this app how to make it better.

We are interested in how you use the app, which is why we are video recording your computer screen and asking you to tell me what’s going through your mind as you go. We are also audio-recording our conversation. The recordings will be used only to help us figure out how to improve the app, and it won’t be seen or heard by anyone except the people working on the project.

If you have questions, just ask. I may not be able to answer them right away, since we’re interested in how people do when they don’t have someone sitting next to them, but I will try to answer any questions you still have when we’re done.

**Opening Questions:**

1. This is the landing page for the app. Please give me your initial reactions to this page and its layout.
2. Please give me your impressions about the app’s menu.
3. What do you think of the colors?
4. Without clicking on anything, please describe the buttons you see and what you think they do.
5. If you were exploring the app, what would you click on first?

I’m going to give you five minutes to freely explore this app.

Next, I am going to ask you to complete various tasks in [app name]. Remember that there are no wrong answers. If a task is difficult to complete, then just let us know that the app needs to be improved.

Quit Journey:

1. Record your mood.
2. Program the app to help you whenever you are at your current location by sending support messages.
3. Say the steps you would take to use the smokerlyzer device to measure the level of carbon monoxide in your breath.
4. Access the message library on the app and pull two or three messages that you like.
5. Find the game Fruit Squish and play it briefly.
6. Redeem a coupon of your choice.

QuitGuide:

1. Find out whether this app contains information about quitting smoking.
2. Set up a quit date a week from now.
3. Contact the quitline on this app.
4. Record your current level of craving for a cigarette.
5. Find information on distractions from cravings in this app.
6. Submit a personal reason for quitting.
7. Program the app to “help” you at a specific location by sending support messages when you are nearby.
8. Stored in the app is the number of days a previous user was smoke free, and the minutes and dollars the user saved by being smoke free. Find these numbers.

**After each task, ask:**

1. What was easy/difficult about this task?
2. Would you use this feature in your quit journey? Why or why not?
3. How helpful is this feature? Was it difficult to understand?
4. What do you think should be changed? What else do you think might be useful?
5. What did you think about the layout of this section?

**Post-Test Questions:**

1. Would you use this app? Why or why not?
2. Do you have any last comments about the app or your experiences with it?
3. On a scale of 1 to 5, with one being strongly disagree and five being strongly agree, please tell me how much you agree with the following statements:
   1. System Usability Scale
      1. I think that I would like to use this app frequently.
      2. I found this app unnecessarily complex.
      3. I thought the app was easy to use.
      4. I think that I would need the support of a technical person to be able to use this app.
      5. I found various functions in this app were well integrated.
      6. I thought there was too much inconsistency in this app.
      7. I would imagine that most people would learn to use this app very quickly.
      8. I found this app very cumbersome to use.
      9. I felt very confident using this app.
      10. I needed to learn a lot of things before I could get going with this app.
   2. I intend to continue using this app.
   3. I want to continue using this app rather than discontinue.
   4. I would recommend this app to someone who seeks my advice.
   5. I consider [app name] to be my first choice for smoking cessation apps.
4. How many times would you use the app in the next 2 months if you were to quit smoking?
5. Would you pay for this app? Yes or no.
6. On a scale of 1 to 5, with one being one of the worst apps you’ve ever used and five being one of the best apps you’ve ever used, what is your overall star rating of the app?

**Supplementary Table 1. Semantic domains and theme definitions.**^23-26,41-43^

| **Technology acceptance** |
| --- |
| “Effort expectancy refers to perceived ease of use or effortfulness with which one can navigate smoking cessation mobile applications and their features and seamlessly integrate them in one’s life.” |
| “Facilitating conditions are factors that can aid or impede the uptake or use of smoking cessation mobile applications or their features. These include individual-related (e.g., skills, predispositions, prior experiences) and technical-related (e.g., infrastructure) factors.” |
| “Hedonic motivation refers to perceived fun, pleasure, or enjoyment (or lack thereof) associated with the use of smoking cessation mobile applications and their features.” |
| “Performance expectancy refers to perceived usefulness or helpfulness of smoking cessation mobile applications and their features in achieving desired health goals and behaviors.” |
| “Social influence refers to perceived importance of significant others’ recommendations and approval of using smoking cessation mobile applications and their features.” |
| **Sentiment** |
| “Negative sentiment capture statements or remarks that indicate a sense of disapproval, criticism, or skepticism about any aspect of smoking cessation mobile applications and their features such as their worthiness, utility and impact, time and effort investment, and compatibility with one’s life.” |
| “Neutral sentiment capture statements or remarks that (a) are neither positive or negative in tone, (b) contain an equal number of positive and negative remarks, or (c) are conditional (e.g., positive in nature but dependent on the presence or absence of another factor).” |
| “Positive sentiment capture statements or remarks that indicate a sense of approval, praise, or certainty about any aspect of smoking cessation mobile applications and their features such as their worthiness, utility and impact, time and effort investment, and compatibility with one’s life.” |
| **Usability task** |
| Tasks 1 through 6 for Quit Journey; Tasks 1 through 8 for QuitGuide. |
| **Other** |
| Intent or willingness to use: “Statements reflecting intentions or willingness to use smoking cessation applications or their features in the future.” |
| Suggestions: “Statements concerned with improvements, modifications, or additions to smoking cessation mobile applications or their features aimed to improve their functionality and/or design.” |

Multi-value coding of quotes was applied across semantic domain, but themes within a semantic domain were mutually exclusive.

**Supplementary Table 2. Participant-level task completion results and task duration for Quit Journey and QuitGuide.**

| **App** | **ID** | **Task** | | | | | | | | | | | | | | | | | | | | | | | |
| --- | --- | --- | --- | --- | --- | --- | --- | --- | --- | --- | --- | --- | --- | --- | --- | --- | --- | --- | --- | --- | --- | --- | --- | --- | --- |
|  |  | **1** | | | **2** | | | **3** | | | **4** | | | **5** | | | **6** | | | **7** | | | **8** | | |
|  |  | **O** | **C1** | **C2** | **O** | **C1** | **C2** | **O** | **C1** | **C2** | **O** | **C1** | **C2** | **O** | **C1** | **C2** | **O** | **C1** | **C2** | **O** | **C1** | **C2** | **O** | **C1** | **C2** |
| QuitGuide | P01 | ✓ | 23 | 20 | ✓ | 35 | 37 | X | 104 | 107 | ✓ | 32 | 32 | ✓ | 32 | 33 | ✓ | 1 | 2 | ✓ | 72 | 75 | - | - | - |
|  | P02 | ✓ | 22 | 24 | ✓ | 24 | 24 | X | 44 | 54 | ✓ | 12 | 33 | ✓ | 54 | 57 | ✓ | 56 | 55 | ✓ | 17 | 20 | ✓ | 6 | 8 |
|  | P03 | ✓ | 6 | 6 | ✓ | 31 | 39 | X | 49 | 51 | ✓ | 19 | 20 | X | 49 | 59 | X | 28 | 29 | ✓ | 79 | 62 | ✓ | 1 | 3 |
|  | P04 | ✓ | 14 | 12 | ✓ | 38 | 36 | X | 81 | 80 | ✓ | 33 | 34 | ✓ | 19 | 21 | ✓ | 3 | 5 | ✓ | 48 | 49 | ✓ | 5 | 6 |
|  | P05^*^ | - | - | - | - | - | - | - | - | - | - | - | - | - | - | - | - | - | - | - | - | - | - | - | - |
| Quit Journey | P06 | ✓ | 14 | 15 | X | 48 | 49 | ✓ | 27 | 27 | ✓ | 7 | 9 | ✓ | 16 | 14 | ✓ | 8 | 8 |  |  |  |  |  |  |
|  | P07 | ✓ | 21 | 21 | X | 58 | 53 | X | 45 | 44 | X | 72 | 71 | ✓ | 40 | 43 | ✓ | 14 | 16 |  |  |  |  |  |  |
|  | P08 | ✓ | 102 | 104 | X | 23 | 27 | ✓ | 79 | 90 | X | 252 | 253 | X | 66 | 68 | X | 66 | 68 |  |  |  |  |  |  |
|  | P09 | - | - | - | X | 150 | 148 | X | 101 | 101 | X | 32 | 37 | ✓ | 27 | 28 | X | 145 | 141 |  |  |  |  |  |  |
|  | P10 | ✓ | 66 | 64 | ✓ | 38 | 40 | ✓ | 101 | 101 | X | 63 | 66 | ✓ | 37 | 38 | ✓ | 48 | 51 |  |  |  |  |  |  |

Time on task measured in seconds.  ^*^Recording was lost for P05.
Some participants did not attempt all tasks due to lost recordings or skipped tasks (e.g., time restraints).
ID = Identification number, O = Outcome, ✓ = Task success, X = Task failure, C1 = Coder 1 (MW), C2 = Coder 2 (GZ).

**Supplementary Table 3.** **Themes and illustrative quotes of smokers’ perceptions of Quit Journey.**

| **Task** | **Theme** | **Quotations** | **Sentiment** |
| --- | --- | --- | --- |
| 1 | EE | P08: The difficult section [for tracking mood] was the, “[Help me] next time I’m here” part of it, just because … I didn't know … what exactly they were talking about. I feel like it was … a little vague, but … since I kind of guessed a little bit that they were … talking about … next time I'm feeling this way to help, instead of like next time I’m on the app … help out. I don't think it's like … [a] contact us type thing. I think it's more of like, oh, we'll send you a notification next time this occurs. | Negative |
|  | EE | P10: The easiest [thing about the mood tracking] is knowing exactly where it's gonna be in, the big green button, and the most difficult or confusing part would be the mood or the craving part. I would open this up to a different detail, or just leave it blank … That my mood and slipping are two different things, it’s just what I was saying. | Neutral |
|  | EE | P06: I think after kind of playing around with the app a little bit, it would be very easy. Like, after just doing what I did pretty much and clicking around, I think it'd be easy to navigate. | Positive |
|  | EE | P07: [The mood tracking] was pretty straightforward, I'd assume. On a phone … you can either press on whatever number you feel, or you can also slide that circle back and forth, so it's easier to kind of get to where you want to get to, but yeah, it was pretty straightforward. | Positive |
|  | EE | P10: [The mood tracking layout is] self-explanatory. Really user-friendly … Giant text, happy face, frowny face. They got words of help at the top. Everything’s just big and in your face. | Positive |
|  | FC | P10: To me no, [I wouldn’t use the mood tracking] because … I'm bad at doing something on a daily basis for, concurrently day in, day out, I can’t just stick behind an app like this.^*^ | Negative |
|  | PE | P08: I think I would have to try it first. To be honest … I don't think [that I would use the mood tracking] … I think that section would be a part that I would … skip through maybe a few times just because it's … not something that's … immediate or … it's not something you really get something out of … you kind of record how you're feeling and then it just goes away, rather than … some of the other features where it'll … give you something, it'll give you like a quote or it'll give you like … a challenge.^*^ | Negative |
|  | PE | P07: No, [I wouldn’t use the mood tracking] just because if I'd be using this app, I’d be using it simply to have an app that can log the specifics of my quitting in terms of how long it's been, how many I haven’t smoked, and how much money I've saved … I wouldn't use an app like this to help with my mood or to work on the psychological aspect of quitting, I’d more so just want to have the app to motivate myself by looking at how long it's been or how much money I've saved, but … I'm not confident that stating my mood on an app like this would actually help improve my mood at all … If you gave me tips or something like that, I just don’t think it’d be helpful. That's my assumption.^*^ | Negative |
|  | PE | P07: Personally, no [I don’t think mood tracking would be useful], just because … I think everyone has their own triggers. There's some things that will trigger people to smoke that won't trigger others, and at the same time, again, I just don't know how it would help me to tell an app what my triggers are if I already know them myself. It’s not like the app can … be aware if I'm not using it whether or not I'm currently … triggered to smoke … It wouldn't be … recording me or know … when this person goes to this location, they normally get a trigger to smoke. I can't imagine the app would be able to detect that and like try to help me not smoke in that moment. | Negative |
|  | PE | P06: Yes, I would [use the mood tracking]. I think that the biggest thing for quitting smoking is identifying triggers. I think that's super important and to know certain times of day or how I'm feeling could be really important to trying to prevent that emotion or feeling from the beginning.^*^ | Positive |
|  | NA | P06: Other than the color, [the mood tracking layout] just doesn't look appealing, I guess it looks kinda plain. | Negative |
|  | NA | P07: [The mood tracking page] feels a little cluttered to be honest with you, but … maybe it will look different on a phone compared to a PC … I could see how it could be possible for somebody to accidentally press the wrong button just because they're all so close to each other … There's very little empty space … it's pretty filled up I would say. | Negative |
|  | NA | P08: I think the layout [for the mood tracking] is nice … that's something I do really like about it … how everything’s laid out. I think it's all … pretty clean. | Positive |
| 2 | EE | P06: It's not an obvious place to go [to access the personalized support]. | Negative |
|  | EE | P07: I never could find… I remember seeing the location, something about location on this app at one point, but I can't remember what page it was on … If there’s nothing about your location on the homepage, and there's nothing about my location in my smoking details, which would be the first place … I’d assume that’s where something about my location information would be … Anything I selected didn't have anything about my location, and I can't remember where it was. | Negative |
|  | EE | P07: The only thing [about the personalized support layout] … Everything kind of looks the same because of the same color scheme, which is nothing but black and white, so it's hard to remember exactly, visually what each thing to click on brought up, because then they all kind of bring up a similar looking menu of some sort. | Negative |
|  | EE | P07: If I could, I’d probably turn off any notification settings in that, so that I could, if I want to use the app … use it, but I don't want to be getting notifications with like motivational messages throughout the day. That would annoy me. | Negative |
|  | EE | P10: You're explaining to get support messages at a specific location, and this … is completely different than support messages. This is my mood [tracking], and if I'm here to get … help at this location, not… [the] advice [tab]? You think it’d be advice and get support messages from here … Or the notification tab … and sign up for support messages via e-mail or text? | Negative |
|  | EE | P06: Now that I'm looking like back, it's pretty obvious [to use the personalized support], but at the time … I think it's just the typical learning curve that anybody would have with any app, and it takes a little while to click on everything and kind of see what it does. But I don't think that there's anything in particular that … could’ve been different that would have made it easier for me to do. | Neutral |
|  | EE | P08: I think … this section [with the personalized support] came pretty easy, just because it's something that … most people already know about in … other apps, like a lot of it is … already programmed in there just to … draw attention to the app but also to … stay on top of your own tasks. | Positive |
|  | EE | P08: The layout [for the personalized support] is all pretty simple and easy going. I think … it doesn't make it too difficult. | Positive |
|  | EE | P06: It was easy [to follow the steps for the personalized support], and … like I said, now that I know where it is, it's easy enough. It's two buttons. So, I mean two things to click on. So, it's easy enough. | Positive |
|  | EE | P10: [Setting up the personalized support was] easy because it's right there, like I said earlier, being advice. | Positive |
|  | EE | P10: I mean, [the layout for the personalized support is] the same thing. As I said before, it's right here it's in your face. You got your smiley face, frowny face. I mean, it's user-friendly. | Positive |
|  | PE | P07: No, I wouldn't [use the personalized support] … just the thought … [of] getting like automated texts sent to me … I don’t know how to put it more politely, but … it’s not real. It's having a robot send me automated texts, like generic messages that it sends to every user. That wouldn't motivate me … it's a robot talking to me, it's not going to help me quit smoking at all. It’s just gonna feel like spam.^*^ | Negative |
|  | PE | P10: No, I wouldn’t [use the personalized support], not this part, but maybe the advice part that you’re explaining … Well, I wouldn't do this part but the part that you're explaining where I'm going to get advice messages is the part I'd probably get into because notifications pop down from my phone all the time, and maybe one sparked my interest. I want to click and go and read this advice part, but … I do not want to get bugged constantly if I'm going to work with this or at home all the time. I wouldn’t do this part.^*^ | Negative |
|  | PE | P08: I guess you would just go to the notifications and … turn on where you’d want to be notified, and looks like you can get the location also, which is just … like a pretty regular feature in a lot of apps, I think, to … have … the notifications section … I think it's a little different that you can have … a location in this one … because it's … smoking-related, and this might be … somewhere you can go to … you know you won’t smoke or something. So, maybe you'll go here and it's like, ok, this is … somewhere where I know … I won’t feel … the urge to smoke or have that same feeling. | Positive |
|  | PE | P06: Yeah, [I would use the personalized support]. So, say I’m at work, and on my break, I usually go out and smoke. I think that would be useful to have … Text me notifications … little tips or something when I'm in that location or at certain times. I think the times would be good. So, say, I go on lunch break every day at 12 o'clock. Having something at 12 o'clock, like, hey, you know, you've saved X amount of dollars already or … you're this close to your goal.^*^ | Positive |
|  | PE | P09: Maybe … that [help me next time I’m here button] is basically saying … let's say I'm at work, and it knows my location, so maybe it knows that … maybe I get smoke breaks because I've been … recording that I smoke around this time. So, when I'm maybe in that location, I am given like a prompt, like, ok, now, I feel like it only because of that location thing, but next time I'm here if it only said help me next time I'm here, and didn't have that location thing, I would assume that … I would just be getting messages … it wouldn't have to do with my location itself. But that location thing, registered in my brain as … it's automatically knowing my phone is on, and location is on, and my phone is going to help me next time I'm recorded being in that particular location. | Positive |
|  | PE | P09: I think I would [use the personalized support], because I notice I have the tendency to go outside and … I'm going outside to smoke. So, if my phone knew, ok, I've moved from maybe a location that I’ve recorded now, that this is a place that I smoke, if it knows I'm there, it's almost like a warning, like, hey … your support is here now. So … hopefully, you're not going to smoke. So, I would put that on so that it's a reminder on my phone, like I wouldn't want the app to go to waste and just sit around and only open when I'm opening it. So, once I put that notification on, it'd be good to maybe get … a notification, almost like a text message, saying … maybe you are in the place where you smoke … normally, and now you don't need to be smoking in that place, maybe let's try to … do something or do this … It’d prompt me to do something, but … I think I would put that on because I'd want to see how much … the app could help me when I'm not automatically in the app and logged in the app.^*^ | Positive |
|  | PE | P09: Yes, [tracking my triggers would be useful] because I would kinda start to see either, am I making excuses? Or … is there something I need [to] change in the pattern of my health? Because maybe the cigarettes are just 10 minutes of blank thoughts and nicotine and smoke and tobacco, and then I go back to being sleep deprived and tired, and so … if I'm literally saying … what triggered me was I was around someone, and there was smoking, if I keep typing I was around someone [when] I’m smoking, then basically all I'm doing is digressing and not helping myself … compared to … I woke up at night and … I didn't have any more sleep, so I smoked. So, that's just me choosing to smoke and be idle instead of going right back to bed. | Positive |
|  | PE | P09: I can basically type a paragraph [about my craving] or I could type … like … I had little amount of rest, so I woke up agitated and wanted a quick release or relaxation time … I would … basically throw it all out there. I wouldn't just write out two words. | Positive |
|  | NA | P09: [The] we are here to help [label at the top of the page] is … kinda bland and it's encouraging though, but … I don't really look at it as much as … I thought I would. | Neutral |
|  | NA | P09: Although they're all blue [buttons on this page], like the first one is a good … navy blue, but then the bottom ones are like … how to quit doesn't really give me the first thing to click. I guess I'd rather go to record, get a tip, take a challenge, play a game, compared to pressing [the white] how to quit [button]. | Neutral |
|  | NA | P07: There were two things underneath [the mood tracking] saying … help me next time [I’m here and tomorrow]. I'm not really sure why I’d need help doing that. | Neutral |
|  | NA | P08: I think just … the notifications section [for the personalized support] is nice, it's pretty, it’s got a pretty simple layout. | Positive |
|  | NA | P08: Yeah, [I would use the personalized support]. I think … that's awesome. I think it's something that not a lot of other apps do. I think they have the time, and they use that for … most of them, but I think using the location is … a good feature also.^*^ | Positive |
|  | NA | P06: I think [the personalized support layout] looks good. | Positive |
| 3 | EE | P06: Breathe in for 15 seconds, so maybe breathe into [the] device. You know breathe in for 15 seconds, I don't know. I feel like that just could be worded better. | Negative |
|  | EE | P07: I don't have a medical background. I have a vague understanding of this type of thing … is that what PPM stands for is parts per million? Like it's measuring your carbon monoxide levels? … I would assume so, but I mean … I'd assume the majority [of] people are not familiar with that unit of measurement, and so to get that number would mean like absolutely nothing to them, because they don't know, they've never measured it before. So, if they saw a certain number … they wouldn't even be able to recognize whether it's good or bad or normal until they kept using it a lot. But still … if you told someone like, hey … your parts per million is at 947, they wouldn't know what that means … whether … that's a big deal … It's a unit of measurement that most people don't understand, I’d guess. I'd assume most people … aren’t familiar with that. | Negative |
|  | EE | P09: I don't know what PPM means [on the carbon monoxide monitoring page]. | Negative |
|  | EE | P09: I [tried] to follow the prompt as hard as I could, and then it would tell me [my]m current reading and average. That's confusing … it’s basically … telling me how much carbon monoxide I have … I guess because I'm not too familiar with, I didn't know it was invisible, to the sense that it would not be detected if my phone can detect it. It's basically telling me … it's kinda high, it looks high to me. Like, 947 seems kind of high. | Negative |
|  | EE | P10: I don't understand what it's reading. That's the difficult part [about the carbon monoxide monitoring]. | Negative |
|  | EE | P10: Oh, you have to connect [the smokerlyzer]. See, that would be awkward … That'd be a lot better with Bluetooth. I dunno why you would always have to connect it to a cord … I dunno, that's not really too friendly. I think that would definitely not get a lot of users to use it just for that fact, honestly, because everything is Bluetooth or Wi-Fi nowadays. | Negative |
|  | EE | P08: I think it's nice that [the smokerlyzer is] portable and it has … like a USB drive where you can just plug it in and use it with your phone. But I don't know how many people really … want to carry around a [smokerlyzer] all the time. | Neutral |
|  | EE | P07: [The carbon monoxide monitoring layout] all seems pretty straightforward. Again, I'm just not a fan of like the color scheme … it feels kind of like a sterile dull color, it just keeps reminding me of … the default Microsoft Office thing with … these blues. But … in terms of … usability, the layout’s fine. If all I wanted to know by going to this section of the app, is to know what this level was, yeah, everything's great. I like the fact that at the bottom, you can get … a previous reading to compare and contrast the two. Yeah, pretty straightforward. I like it. | Neutral |
|  | EE | P07: I think [the carbon monoxide monitoring would] be pretty easy. The only thing I can imagine that would be a pain is … every once in a while, wireless devices can be hard to connect via Bluetooth. Sometimes they work, sometimes they don’t, so as long as it worked every time, I'd imagine it’d be pretty straightforward. | Neutral |
|  | EE | P06: It was easy [to use the carbon monoxide monitoring]. It was really easy. I liked the location of it, too. I like how it is in this little drop-down menu, I like how it’s at the top of the drop-down menu … It's right at the top. | Positive |
|  | EE | P06: So, I think having it user-friendly without that [smokerlyzer], so … put … at a very obvious place, like, here it is, it's right here, easy to get to, but without being like, hey, in your face, you need to buy this. But other than that, it looks very, very easy to connect. The little pictures, like everything, it just looks very easy. | Positive |
|  | EE | P08: I feel like … [the carbon monoxide monitoring is] fairly simple. It doesn't take a lot of work … I think it was pretty simple. | Positive |
|  | EE | P08: I think [the carbon monoxide monitoring layout is] nice because … [it] just says plug in your smokerlyzer to your device and then turn the volume all the way up. So, yeah, it seems like the layout is pretty nice … it’s got like similar colors, it's got … the words … you need to see are in bold and … I feel like that's the most important … especially for something like this, where I feel like it's pretty simple. It's a pretty simple … tutorial on how to use the smokerlyzer. | Positive |
|  | EE | P08: I feel like [the carbon monoxide monitoring instructions] were pretty easy to understand. | Positive |
|  | EE | P07: Yeah, [the carbon monoxide monitoring instructions were] completely clear. I mean if someone did have this device … I would say that everything is clear in terms of how to properly do it. Nothing’s confusing or misleading. I think it's very straightforward, this aspect of it, yeah. | Positive |
|  | EE | P10: The easy part [about the carbon monoxide monitoring] is … it's self-explanatory. You're going right through it. | Positive |
|  | EE | P10: Oh, yeah, definitely [I would use the carbon monoxide monitoring] … It's in your pocket and if I don't have to open up my app all the time and I just pull that thing out … you got those vapors out why can’t you blow through one of those [smokerlyzers], you know? So, keep one of those in your pocket, and … this automatically records it for you.^*^ | Positive |
|  | EE | P10: No, [the carbon monoxide monitoring instructions were not difficult] I mean, it just says blow through it for a certain amount of time. | Positive |
|  | EE | P10: [The carbon monoxide monitoring is] easy and self-explanatory. | Positive |
|  | FC | P08: This [carbon monoxide monitoring] is kind of like confusing to me, just because I've never really worked with one of these [smokerlyzers] before. | Negative |
|  | FC | P08: I'm not sure, actually, I've … never really used one of these [smokerlyzers]. | Neutral |
|  | FC | P06: I think what's very important about this app is that it's usable without this [smokerlyzer] device. I think that's a really important thing because, honestly … I feel like not a lot of people are going to buy it, honestly. | Neutral |
|  | FC | P07: I would use [the carbon monoxide monitoring] just because … it would come down to this device … If it somehow came free with the app, I would absolutely use it just out of curiosity, just because … I'm the type of person that likes testing things and getting an exact number on something. So, I actually could compare and contrast, so yesterday my number was this, today it's this. But, if I had to pay for this device no, because I would never buy it, I would never buy something that would measure that, and if I did … to be honest with you, I'm gonna assume that there's other versions of this device that are probably cheaper that just don’t come with an app. So, if I really wanted to know this number, I’d just buy the cheapest one I could find on Amazon, and then log it myself if I cared that much.^*^ | Neutral |
|  | FC | P06: I don't think I would use [the carbon monoxide monitoring] personally … I just feel like it doesn't seem like I need it …I just don't feel like I would need it.^*^ | Neutral |
|  | FC | P07: Especially something like this [smokerlyzer], I would never pay money. I would never buy a device just to connect to an app like this … unless it was absurdly cheap, then maybe. But most likely, I'm gonna assume, something like this is not gonna be that cheap.^*^ | Neutral |
|  | FC | P08: I guess [the smokerlyzer] could be nice if it worked for like all phones or if … the USB was … compatible with … any type of phone. Then it might be nice because then … everyone can use it, like people who are … low-income and can't afford … a super expensive phone that … has … all these features … Then they could … get this app and use this smokerlyzer for that, but I'm sure if this was … put into place, then I think there would be … several different kinds of … USB drives that would work with this. | Positive |
|  | PE | P10: [The smokerlyzer information is] not helpful at all, because I don't know what it’s referencing to … Now if I knew what it was referencing … then maybe I'd be more educated and wanted to know more? So … I want to keep down whatever PPM, parts per million, you're trying to keep down. | Negative |
|  | PE | P08: I think this [carbon monoxide monitoring] is a pretty cool feature just because it's portable and … it’s something that’ll … kind of keep you in check with yourself and … know the right thing to do at the right time. | Positive |
|  | PE | P09: I would want to [use the carbon monoxide monitoring] because I would want to … be able to show somebody … see my … life, my health is changing, I can show it to you … proof-wise. Or, I would want to encourage somebody to quit as well because I can tell them, with proof and data … here, look, this is what's actually changing, I have records … showing you that it's not that hard to quit and look how much it’s benefiting me in [this] amount of days, hopefully showing that, although I might have started two days ago, things are changing, compared to, oh, you think you quit one day, and it's not worth it those 2, 3 days that you quit or that you’ve refrained from smoking. So, I would really wanna use it because it kind of seems cool to have something assisting me other than just the app … It would keep me occupied, it would be like okay, I don't want to check it too often, but when I do check it, hopefully … I see progress instead of just nothing happening because then it would help me understand, am I helping myself at all? … Are things changing? Yeah, but I would want to use it … I wouldn't want to just have the app and then it has a feature like that that could be mailed to me or that I can buy, and I don't use it. It would be kind of pointless.^*^ | Positive |
|  | PE | P09: This carbon monoxide … I think it's because now I've been trying to quit, I'm thinking from the perspective … is carbon monoxide affecting me right now … that's why I'm craving … So, I need to check … what that level is and maybe that's why … Then, I can maybe say that's kinda why I'm feeling the way I'm feeling. | Positive |
|  | NA | P09: The blank background of it being white just kinda makes this [side menu] look like … I'm on a web browser, compared to actually being on an app. | Negative |
|  | NA | P09: The smokerlyzer test … little symbol for it kinda makes me think … like waves and like radiation. It didn't really make me think … like I’m taking a test … It makes it seem like I'm gonna to do something physically. | Neutral |
|  | NA | P10: [The carbon monoxide monitoring layout] really attracts you besides that cord. Oh, my gosh, that cord bugs me. I dunno if it bugs you. | Neutral |
|  | NA | P08: I guess I'd be open to testing [the carbon monoxide monitoring] out and seeing how it works.^*^ | Positive |
|  | NA | P06: I actually like this part of [the app]. I think that this [carbon monoxide monitoring feature] is well done. I like how it has … the drop-down seconds, how it shows how long it is … It looks good. It looks good to me. | Positive |
|  | NA | P10: Oh, it was good, good layout [for the carbon monoxide monitoring] … I mean, just the color. Color goes with everything, it’s nice, it’s simple. | Positive |
| 4 | EE | P08: I think I'm a little confused with this section of the app [with the message library]. Yeah, I guess … it seems a little jumbled up, sort of. | Negative |
|  | EE | P08: Not exactly sure how to … pull these [messages], or I think you could just … like them but I’m not exactly sure. | Negative |
|  | EE | P08: I feel like on this [message] … I'm trying to scroll and it's just … not doing much … My original thought was to scroll, so I clicked on my tips … thinking there would be more in there … and then I just scrolled down and then … I didn't see any more. I'll click on more tips here and see, but yeah, it still seems like a pretty confusing section. | Negative |
|  | EE | P06: I guess it was kinda difficult [to use the message library] because I didn't know how to go about it right away. | Negative |
|  | EE | P06: Yeah, I can’t like scroll through [the messages]. | Negative |
|  | EE | P07: What was difficult about [using the message library] is that I couldn’t find it at all … I feel like something as basic as messages, that should be something that you should be able to click like in the corner of the home screen, but the fact that I can't find any sort of anything referring to messages or a message library … it’s not good. | Negative |
|  | EE | P10: [What was difficult was] not knowing if I'm in the right place … because I don't see any messages, these are tips or advice that they're explaining. Messages would be more so … messages coming through the app, I guess, from you guys, messages or messages from different users coming to you directly, but advice would be definitely different than messages. | Negative |
|  | EE | P10: Ok, I'm stumped I have no idea where the message library is. | Negative |
|  | EE | P08: I think it was pretty easy to find … the advice section, like once you get here it's a little difficult to move around … kind of like be comfortable in this section of the app just because it's hard to … scroll through, like I said, and kind of get your way around. | Neutral |
|  | EE | P08: I think [the message library layout] looks really good, it's just the scrolling part of it and like the getting to see it, is … the difficult part. | Neutral |
|  | EE | P06: No. I don't think so [that the message library was difficult to understand]. | Positive |
|  | EE | P09: Nothing here [in the message library] was difficult to understand. | Positive |
|  | EE | P09: I’ve understood what [the message] said, and I appreciate what it said. | Positive |
|  | PE | P07: No, [I wouldn’t use the message library] … If I was using an app to quit smoking … I would have zero interest in … meeting or talking to people or sharing stuff with people. It’d be something I’d kinda want to do more by myself and keep private … I think there's gonna be different people who might say yes to that question, but for me, personally, hearing … success stories or motivational messages from strangers on the internet, I could care less about that type of thing. It wouldn’t help me at all. I would never click on it.^*^ | Negative |
|  | PE | P08: This [message] says make half of every meal fruits and vegetables, which is … very positive it’s … reminding you to … always put … fruits and like greens … like what you eat just to … make yourself feel better. | Positive |
|  | PE | P08: I think the information they have provided or the tips that they give you or … that are shown are super helpful … I think it's all great. | Positive |
|  | PE | P09: I kinda wanted to go on [reading the message] where it said “…” after craving … even this morning I was … informed that the fact that I haven't smoked at all … I started to feel some type of fatigue in my body, so now when I read this [message] … it was … confirmation for me understanding why I maybe feel like I'm struggling and not really like rewarded now that I've stopped smoking for a day … Yeah, so it was helpful. | Positive |
|  | PE | P06: I like the, “you're not alone,” [message] … I think that that's a really important thing to get across. Like, you're not alone in this, [it] is a journey and … it is hard, it's not easy. | Positive |
|  | PE | P09: I would want to read through [this message library] because it's almost like I take my time out to do that on Instagram a lot with people's … captions. So, if it's something … that would help me understand the reasons I'm feeling the way I'm feeling, I would enjoy that, and then I would like to put hearts on them to keep myself … reminded of the ones that I can keep going through, or just show appreciation for the fact that it actually helped me, instead of just reading it and moving forward.^*^ | Positive |
|  | PE | P10: Helpful, because [the message library] would help me wanna know what other people are saying because you can also put your tips in. | Positive |
|  | NA | P06: I just don't like the color [of the message library]. | Negative |
|  | NA | P09: Then [for] advice, unfortunately, the light bulb [icon] was the thing that kinda, unless maybe it's gonna to light up and then it's yellow or something, then I know I have a new message, the color that it is right there doesn't really automatically … make me think messages. | Negative |
|  | NA | P06: Possibly [I would use the message library] … I guess it depends on the content of the messages … Maybe more like inspirational quotes, I guess, but maybe more like typical inspirational quotes … like, one day at a time, like, even just corny stuff like that.^*^ | Neutral |
|  | NA | P10: Yes, [I would use the message library] if they came down in a notification … but you wouldn’t catch me just openin’ up the app out of nowhere just go to this tab and go look at them. It would have to pop down, pique my interest, then I'd go into the app, and with this part, right here, definitely makes me want to read what's over here.^*^ | Neutral |
|  | NA | P09: Now [the messages are] going sideways, I guess because I'm so used to scrolling up and down, sideways is kind of like I would have to get used to not skipping too fast. | Neutral |
|  | NA | P06: I like these challenges though, write your favorite motivational or inspirational quote in a notebook. | Positive |
|  | NA | P06: I actually really like the layout [of the message library]. | Positive |
|  | NA | P10: I like [the message library layout] because you can swipe, obviously, and go through ‘em, you know. | Positive |
| 5 | EE | P08: I'm actually not sure where this game would be … Yeah, it's a little confusing, this part … Just because I don't think there's … anything that … really shows that there would be a game on here … It's pretty … hard to see that there would be a game on here. | Negative |
|  | EE | P06: It doesn't say the name of the game anywhere. | Negative |
|  | EE | P07: Yeah, I have absolutely no idea what each icon meant [in the game]. | Negative |
|  | EE | P07: [The game layout is] fine, I guess. Kinda what anything you want out of the game in terms of something like this. It tells you how to play it, and then it tells you your score. It seems pretty basic. Pretty straightforward. | Neutral |
|  | EE | P08: I think it was fairly easy [to understand how to play the game] … I mean, I would have to play it a few times, but I think … from like looking at it first, it was pretty easy. | Positive |
|  | EE | P06: It was easy. It's definitely a simple game. | Positive |
|  | EE | P06: It was easy to know where [the game] was, I guess. | Positive |
|  | EE | P06: Oh, no [the game instructions were not difficult to understand], not at all. | Positive |
|  | EE | P06: [The game layout] looks fine to me. It looks like a typical game that it looks easy enough to understand. | Positive |
|  | EE | P07: [The game] seems pretty easy, seems to follow … that genre of mobile games … that Candy Crush or … Fruit Ninja … just kind of like quick pressing on things and clear off the screen, like a reflex-type game … Seems pretty basic, pretty easy to understand once you play it a couple of times, you just understand the entire premise is to touch nothing except for oranges. | Positive |
|  | EE | P07: The [game’s] instructions were pretty much completely straightforward, it was pretty much just one line of text saying, press the oranges, something along those lines. So, yeah, the instructions were clear. | Positive |
|  | EE | P09: I like that … I knew that the game would help me during my cravings, I knew that I had to press [I’m] craving because it would like save me … I pressed play a game, ‘cause that's how I'm feeling right now, so it was very easy. I knew where I was gonna get it because I do remember when we did go through the prompt … that I saw it … Then … touch the oranges it … literally told me touch the oranges … So, I was pressing all the oranges and all the fruits I could find just to avoid the cigarette packages and … not understanding that maybe that was gonna help my points … I look at that now and say, ok … I'm glad that that was something I skipped because that's how bad I was craving. So, maybe that should make me realize that I'm not even reading directions, and although it … didn't say ready, set, go … it was very easy … Even though it didn’t say … ready, set, go, I guess it’s because they don't really have time or patience, maybe … It's good to help me there … like SOS … Now I understand the game because I read the directions. That's how I would … probably succeed and not get frustrated playing the game and getting less amount of points. | Positive |
|  | EE | P10: Easiest [part about the game] would be clicking or tapping on your phone. | Positive |
|  | FC | P06: I'm gonna play a Candy Crush style game over this … Honestly … if this was the only game, I probably wouldn't play it. I would probably play a different game on my phone, like a different app.^*^ | Negative |
|  | FC | P07: No, [I wouldn’t use the game] because I personally don't play mobile games, and then even if I did … if I wanted to play a game to distract myself from a nicotine craving, I would just play a game that I think is better than this … No disrespect to whoever made it, but I mean, it's clearly … not a superior game in terms of this genre of mobile games … There's gotta be better ones out there that you can just download independently of this app and play yourself if you need a distraction. I would never go into the app to play this basic of a game.^*^ | Negative |
|  | FC | P08: I think [the game] would be fun for a lot of people, just because it … looks similar to like that app, Fruit Ninja, which is … really popular and … a lot of people really like that game. | Positive |
|  | FC | P08: I think … how they made [the game] looks pretty nice … I think the layout is really nice, actually. Like I said, it's pretty similar to Fruit Ninja, which is … another game. Looks like it's similar to the game, not necessarily the app. It’s very different from the app, but … when you're talking about the game, I think it's made very well. I think it's … pretty cool. | Positive |
|  | HM | P06: No, [I wouldn’t use this game] … It doesn't seem enjoyable to me.^*^ | Negative |
|  | HM | P06: I just don't think [the game is] fun because of how easy it is. | Negative |
|  | HM | P07: [The game] gets old pretty quick if all you do is click on oranges. | Negative |
|  | HM | P07: I can't imagine anyone, I mean, I played this game one time and I would never be inclined to play it ever again. I can't imagine anyone would be [enthralled] by this game after more than 30 seconds. | Negative |
|  | HM | P08: I feel like this would be a pretty fun game for people. | Positive |
|  | PE | P06: I just don't like the pack of cigarettes [image], I really don't like that because … I'm playing a game to get my mind off smoking. I don't want to see a pack of cigarettes. | Negative |
|  | PE | P10: Hardest [part about the game] … you see those cigarettes? … That’d make somebody want a cigarette, I wouldn’t have a cigarette in here. If you do reference a cigarette, reference [it] in a negative way instead of a good way, because that looks like you're pulling out a cigarette and you’re about to light that sucker … I dislike the cigarettes. | Negative |
|  | PE | P10: It's [giving me] negative [points] …. on a strawberry. What? I am upset and I want a cigarette. Did you see that? It made me want to smoke, this app. | Negative |
|  | PE | P08: I think so, [I would use the game]. I think I would try it. I would at least try it … because I'm pretty open to … trying … new things when it comes to … apps and stuff like that … if it's … a feature where they think it would … help somebody and … it doesn't harm anything then there's no reason not to try it, I feel like.^*^ | Positive |
|  | PE | P09: I haven't smoked … and I didn't … have a smoke-free day, I'm literally craving. So, I knew I needed to play a game to keep myself distracted, and right now, literally, [I’m] hoping that it would work | Positive |
|  | NA | P10: [The game is] basic. This is something that I would expect the iPhone 1’s first games to be. | Negative |
| 6 | EE | P07: What's difficult about [the coupons]? Just that right now again, it's because [the app is] not a finished product, it all looks cluttered and … there's no actual rewards shown. It's all generic and I can't actually scroll through it, so what’s difficult about it is I can’t actually explore this aspect of the app. | Negative |
|  | EE | P07: I don't recall seeing any instructions for redeeming coupons … I guess it's non-existent from what I’ve seen. I don't remember anything showing me how to do it. I just remember finding the screen itself. Again, it's easy to get to the screen, but I don't see any instructions on how to redeem them at all. | Negative |
|  | EE | P10: [What was difficult about using the coupons] not knowing if I’m getting my money, for one, and then I don't know if it approved me. | Negative |
|  | EE | P10: Yes, [the coupon instructions are difficult] because … I have nothing to go off of besides what I'm looking at. | Negative |
|  | EE | P08: I think it was pretty easy [to find the coupons] because … I guess it's … common sense to … a lot of people. | Positive |
|  | EE | P06: It was super easy [to find the coupons]. | Positive |
|  | EE | P07: What was easy about [the coupons] is getting to it. I'm gonna assume that something like this … if you're actually offering … a financial reward to people, they're gonna pretty quickly remember how to get to this screen … I think maybe just like a subconscious thing, you remember where the rewards are. So, getting to it I think is gonna be pretty easy, ‘cause there's … a value in remembering where the rewards screen is. | Positive |
|  | FC | P06: I knew from my knowledge … of apps in general, I knew exactly where [the coupons] would be … and how to how to access it. It was right where I would think it is, based on my knowledge of cell phones. | Positive |
|  | PE | P10: Oh, for sure [I would use the coupons] if it actually worked, if I was actually getting paid out in U.S. dollars for not smoking cigarettes. But … how long does it take to get 20 bucks? … That's the kicker, right there.^*^ | Neutral |
|  | PE | P08: I think that's a good idea, to reward somebody who … didn't smoke or is like trying to … fix their life or do something in that way. I think it's nice to … have … small rewards like that. I think … that's a pretty positive thing to do. | Positive |
|  | PE | P08: If you're like trying to do something better for yourself then … you know you're gonna get helped out … if you do something good, then you're rewarded for it, I guess that's like what this app is trying to convey, I guess. | Positive |
|  | PE | P08: I think I would try [the coupons] … Yeah, definitely … just because … it's like, trying to help you and … it’s like you are smoke free and it gives you … a coupon to … better yourself or better … something else … I think it’s nice. In that way, I think I would try it. It’s not something I'm against.^*^ | Positive |
|  | PE | P06: I think I would [use the coupons]. I think everyone would like the extra motivation … It feels good to accomplish … it's almost like being recognized for your accomplishments.^*^ | Positive |
|  | PE | P07: If it was real, like … this is real money we're talking about, yeah, absolutely I would [use the coupons] … ‘Cause … there's a financial reward in doing so. It's a big motivator for most people, I think, ‘cause it’s actually a real reward and not just … fake internet points.^*^ | Positive |
|  | PE | P10: That [coupon feature] would be really cool and that would be really unique because you don't see companies doing that, and if you can actually pay out money to me for not smoking cigarettes, heck yes, bring that 20 bucks on. Just put it onto my Amazon card. | Positive |
|  | PE | P10: Like, we're just using [the app] to quit smoking cigarettes, and if we get rewarded, like cool … that’s a huge benefit … for sure … It's all about the money. Like, I want to make sure that … I wanna get that money. | Positive |
|  | NA | P07: This “reward link preferred” [graphic] is so big and … everything else on top is somewhat smaller … Maybe it's … just me, but … it looks a little cluttered or kind of … just not finished. | Negative |
|  | NA | P10: [The coupons layout] can be more or less … It just, honestly, depends on what the final product looks like because I'm just looking at a … few simplistic things, just an image. | Neutral |
|  | NA | P08: I think the layout [for the coupons] is pretty nice, it's all got … a similar or like Earth tone layout with … not a lot of text … too cluttered on the screen, so I think … it's nice in that way. | Positive |
|  | NA | P07: I like [the coupon layout], I guess … If it's something like a rewards section of an app, things I want to see when I open it is how much money I have, whether or not it expires obviously, and … my options for redeeming the reward. So, it pretty much has anything I would want out of it. | Positive |
| General | EE | P07: Again, I’m just gonna repeat, the thing that’s most annoying to me … it's really the only thing that’s really annoyed me so far, is every time I want to go back it takes me all the way back to the home page … if I click on the Smoking Details, and then I wanna go back, I want to be brought back to the Smoking Details page, and not all the way back to the homepage. | Negative |
|  | EE | P07: Not sure what the I’m craving thing [on the landing page] means, necessarily. | Negative |
|  | EE | P07: First of all, I don't know what [a] smokerlyzer test is, it’s kind of a complicated word. I’m not really sure what that is. | Negative |
|  | EE | P07: I don't know … the difference between awards and rewards. I guess, awards would just be … milestone things and rewards will be some kind of points you get that you can redeem somehow, I guess. But, again, unless they're of material value, I would not be inclined to ever kind of spend my time working on any sort of point system. | Negative |
|  | EE | P07: I have no idea what this notification thing means [on the notifications page]. I don't know what I'm supposed to be timing or setting a time for here. | Negative |
|  | EE | P07: I can't scroll [in the history page] … this is just a history of what I was using in the app, I guess, and … first thing I noticed if I was on the phone is there's no … back button anywhere, so I’d have to go straight all the way back to [the] home [page] to get anywhere else. | Negative |
|  | EE | P10: I like [the smokerlyzer]. It’s something that I probably wouldn’t use, honestly, myself, because I'm more so a person that is hands-on, I have to do it myself, it’s gonna be cold turkey … I can’t just track stuff like this. Maybe a lot of other people can, and it looks really user-friendly.^*^ | Negative |
|  | EE | P09: Help me next time tomorrow … I’m confused as to what it would mean. | Negative |
|  | EE | P10: Oh, breath test is that more? Like how would it … analyze how much nicotine you are smoking? How many cigarettes you’re smoking? Is that like a … breathalyzer unit for alcohol? I dunno. I’m very intrigued on that. | Neutral |
|  | EE | P08: I think … it's a pretty nice app … it's a good layout I think … it just still has like a little bit of working on to do with some sections, but I think overall … it's a pretty easy to use app. | Positive |
|  | EE | P06: I like [the app], and it looks pretty easy to use … it looks nice … I like the background of it. It looks positive. I like the colors of it. | Positive |
|  | EE | P07: [The app] looks pretty easy to use in terms of there's not too many small icons, I don't know what they do. It seems like everything seems to serve a purpose so far. Doesn’t seem too confusing. | Positive |
|  | EE | P10: So, journey, and then you got the mountains in the back. So, obviously, [it] is going to be a long journey and when you go trekking, which I do all the time, then it’s gonna to be a long road in front of you, obviously. I mean the layout, it’s nice, I like it. User-friendly. | Positive |
|  | FC | P07: I would never really be happy about sharing my location just because it's completely irrelevant, so I’d probably see if I could still use the app while also blocking location settings on something like this just because … I don't know why you need my location. | Negative |
|  | FC | P07: At some point if I use … the I slipped today thing, I don't know I've used an app in the past, maybe a couple of years ago, and the one thing I didn't like about it, that I see with this too, is that … when I use an app like this I kinda want to build up. I guess it’s a mental thing, you want to build up the amount of time you’ve saved and that money you've saved, and I feel like when you kind of admit on the app that you maybe smoked a cigarette, some people … maybe aren't inclined to want to admit [that] just because it’ll ruin kind of their … stats on the app. So, I always kind of wondered how honest people would be when it's something like, I slipped today. It sounds almost kind of like a negative. | Negative |
|  | FC | P10: Pre-planning your quit date. I dunno, that’d be more to a lot of other people, I can’t plan something like that. Like I said, I have to [quit] cold turkey. | Negative |
|  | FC | P07: If there were a financial reward, like this reward thing, and I didn't have to buy whatever that smokerlyzer thing is, I would use [the app] just for the financial reward, but if the only way I could get the reward is by using that device, then I would never, never do it.^*^ | Neutral |
|  | FC | P07: I just think … [whether or not I use the app] all comes down to the money thing … If I were just looking at this in the app store [and it cost money], my immediate thought would be, oh, this is just some tech company trying to profit off people trying to smoke, just like a … quick way to get money ‘cause people are like, oh, I want to quit, and then, if I put money into it, then maybe … it’ll motivate me to make sure I definitely don't quit this time. So, it's kind of like people almost putting … collateral up against their trying to quit smoking by being invested in it financially, and I just feel like that's profiting off of peoples’ weaknesses. So … I would just never pay money to try to quit smoking, I just would never do that just on principle.^*^ | Neutral |
|  | FC | P07: I think some of the features are good and I think a reward system that's, like, real is great … If I have to spend money as well … I can see a lot of people who potentially … would use this app if it was free. But the second they found out they had to spend money, they would stop using it or uninstall it. That's my assumption of what … would happen. | Neutral |
|  | FC | P07: I was smoke-free today thing … I'm not the kind of person that's usually going to login every day for something like this, it’s more so a thing that I would just check periodically to kind of get an idea of how long it's been or how much money I’ve saved. I don't know if I would open an app every day just to press, I was smoke-free today, each day. | Neutral |
|  | FC | P09: The amount of time I've been smoking, I didn't even think [this app] would be created. I thought people were more worried about … other things like weight loss. I didn't know there's anything for [smoking] on phones for some reason. | Positive |
|  | FC | P08: Yes, I would try [the smokerlyzer] … just because of its … portability, I guess, and … it’s something new, something I've never tried before … I think it's something a lot of people haven't tried before, so it would be nice to … try it out and see how it works.^*^ | Positive |
|  | HM | P08: Personally, I don't really know how I feel about the challenges … I think they're nice, but they kind of have … a different side to it, where … I don't know if it should necessarily be … something like fun out of it … I think it should be like positive, but I don’t know if it should be … super fun, like quitting smoking should be … like a game or … something that you're just … playing around with. | Neutral |
|  | PE | P06: I don't really like the game. I don't like that. It shows the cigarettes … Maybe something else, but … I feel like that could trigger a craving for someone who's trying to not think about cigarettes. | Negative |
|  | PE | P07: Awards wouldn't really motivate me at all because I'm assuming there's nothing, whatever the award or rewards are, they're not material, so it's … just like an internet points type thing, I really wouldn't care about at all to be honest. | Negative |
|  | PE | P07: I'm not gonna feel inclined to not smoke based off of like an icon in an app … [the history feature is] not going to motivate me at all, but … for some people it might be helpful. But yeah … this type of [feature] isn't gonna motivate me to quit smoking at all. | Negative |
|  | PE | P07: Yeah, this kinda thing doesn't work for me, all these kind of tips [in the message library] … I feel like you've already heard them all … telling me “I'm not alone” in quitting cigarettes, I don't think that would ever have an actual effect on me. Yeah, I mean, I get it, but this isn't the kind of thing I'd want in an app. I don't need … an app to tell me smoking’s bad for me, like I already know that. | Negative |
|  | PE | P06: I feel like this history [feature] is kinda useless. | Negative |
|  | PE | P07: Yeah, I just wouldn’t, playing [a game]—like that wouldn’t help at all with quitting smoking … I can find my own distractions. I don’t need … another app to show me games to try to help … Like someone else, if they wanted to play a game, they would play whatever their favorite game is on their phone … My first thought of oh, I want to … smoke a cigarette, I should distract myself by playing a game wouldn't be to open up an app and play whatever game this app would give me. I would just find my own … something like Candy Crush or whatever they play. | Negative |
|  | PE | P10: Games, sweet. So, touch the oranges. I don't like that because if you're trying to quit smoking cigarettes, you're seeing cigarettes over and over right there, just like in the movies. | Negative |
|  | PE | P07: Yeah, these seem like basic tips [in the quitting tutorial] … I guess for some people that would be helpful … This wouldn't be my kind of thing I would use, at least this aspect of it.^*^ | Neutral |
|  | PE | P07: If [the coupon is] actually $20 off something that I could use, yeah, that'd be great. Definitely, I’d use something like this as long as it's relatively passive, which I assume it would be if all I have to do is everyday log whether or not I’ve smoked, but then also, I'm not really sure … If you're having like a financial reward, for a thing like this … I’d just maybe just lie, just to get $20, no matter what, you know? If I knew that as long as I kept saying that I haven't smoked for a … certain period of time, I would definitely … I'd assume people would just lie in order to get the $20.^*^ | Neutral |
|  | PE | P07: It has pretty much anything I'd want out of a smoking cessation app, just the amount of money I've saved, how long it's been since I've smoked, and the amount of cigarettes I haven't smoked. Again, I'm not sure exactly what the time saved aspect of this thing would mean, but I'm guessing it just means how much time I haven't spent smoking. | Neutral |
|  | PE | P08: I think I would use this app just because of maybe … the I'm craving section, I think that part is … pretty helpful, I think. I'd use that pretty often just when … whenever I feel like … I need to smoke or something like that, or something in that same feeling where I would go and try … a day where … I'm trying to do … better for myself, and I would go, I was smoke-free today … try and track my history … build it up like that, or vice versa, if I slip today and then … you click on that and then see … how many days in a row or how many times you've slipped … So, I guess I would use it for those, but I don't think … I'd really use it for like the advice or … the games or … the challenges and stuff like that.^*^ | Positive |
|  | PE | P08: I think it's nice that it actually does, looking closer at the app, [have it] where you can get a tip on how to not smoke or not crave smoking in the future. I think those are always nice. | Positive |
|  | PE | P06: Yes, I would [use this app] … I think any tool that could help give me an idea of what my triggers are and when I smoke and things like that, where I smoke, I think anything that could be helpful in my quitting journey I would definitely use … I'm excited to see what it's like when it's done … I'm excited to try it out.^*^ | Positive |
|  | PE | P07: I do like … the fact that the second you open [the app], it tells you, kinda the basic stuff. I feel like you’d want to know from an app like this, like how much money you've saved and how long it's been since you quit. | Positive |
|  | NA | P09: The I slipped today … it just kind of looks … not as exciting as I hope, like maybe … [if] it wasn't so orange, maybe more gold. I’d be excited to press it … that's just the first thing I noticed. | Negative |
|  | NA | P06: I think maybe that red I slipped today [button], I think that kinda looks a little harsh. | Negative |
|  | NA | P06: This [smoking details menu] just looks kind of plain … nothing in particular, just looks a little plain, it looks like a lot of white. | Negative |
|  | NA | P07: [The colors on the landing page are] a little dark. The dark blues kind of have like a, not depressing vibe, but it's not as colorful. It kind of seems like they're not very bright colors. They're all kind of dull, except for the red and blue at the bottom where it says I was smoke-free, and I slipped today. Kinda reminds me of like basic … Microsoft Office default colors in a way … Yeah, not as colorful, kinda plain, kind of bland, but, at the same time, I'm not the kind of person who would care about that. I’d care more so about what the app does and how well it works. But … just like a general … artistic standpoint, it's a little dull. | Negative |
|  | NA | P06: [The side menu] looks kind of generic … I think it looks good. | Neutral |
|  | NA | P06: I actually like this whole thing. It looks kinda plain, like just being white, but I feel like that's just because it's … a new app. | Neutral |
|  | NA | P09: I guess maybe the blue of I was smoke-free today … [is] like rewarding pressing it compared to the [red] I slipped today. | Neutral |
|  | NA | P10: Honestly, the advice button is what I clicked first before I even got on the screen sharing. That's where I went … because there's not really much more to click on in the beginning [on the landing page]. There’s no settings, nothing that you can correspond to yourself, you can’t really do anything right here besides three buttons. | Neutral |
|  | NA | P08: I think it's a nice design [for the app]. It's … pretty colorful … you can kind of put your attention in like different sections of the app, like where it says, I was smoke-free today. | Positive |
|  | NA | P08: [The app] looks pretty sleek and clean. | Positive |
|  | NA | P08: I think [the app is] nice, it's pretty simple. It’s got like a nice layout, just black and white … it doesn't look like there's too much to it besides just … a simple layout. | Positive |
|  | NA | P08: The colors are nice … they're kind of like earth tones, they're all like nothing too bright … besides … the little ribbon there [above the smoke-free button] … I think everything is pretty … simple and nothing’s … too … eye-catching, but also shows you … what you want to see in like a nice space, I guess. | Positive |
|  | NA | P06: I like the little logos that [the app] has, and I like the options available, so the time saved, I like that. I like the money saved; I think that's really cool. | Positive |
|  | NA | P06: I like the colors [of the landing page], I think it looks nice. I think it looks positive. | Positive |
|  | NA | P09: The background [of the app] … the colors make it seem serious, like this is like an app that I'm gonna respect like I'm not gonna treat it childlike. | Positive |
|  | NA | P09: It said money saved because that's what I wanted to know … am I going to be saving a lot more once I start quitting? And then … I'm craving … now [that] I look at, it registers like SOS … like help … but at the same time, it wasn't the first thing I looked at. | Positive |
|  | NA | P10: They’re subtle [colors] … green, blue. I mean, that big, red I slipped today [button] … that definitely brings my eye to it … the color layout’s nice. The theme in the background goes great with the actual color layout. | Positive |
|  | NA | P10: When you guys get this done and up, I want one of those units, for sure, and I want the app, I want to try it.^*^ | Positive |
|  | NA | P10: Yes … I would use [the app] because the smokerlyzer and the reward systems, but if you implemented it to the leaderboards and all that other stuff that I've talked about, I mean, I would go to like a 4, 4 and a half [rating], and I definitely would use it.^*^ | Positive |
|  | NA | P10: Then check awards, obviously that's done with the awards thing. So, I mean … it looks nice. | Positive |

Participant identification number appears before each quote for attribution.
^*^Indicates quote references intent/willingness to use.
PE= performance expectancy, EE= Effort expectancy, FC= Facilitating conditions, HM= Hedonic motivation, SI= Social influence, NA= Not applicable.

**Supplementary Table 4.** **Themes and illustrative quotes of smokers’ perceptions of QuitGuide.**

| **Task** | **Theme** | **Quotations** | **Sentiment** |
| --- | --- | --- | --- |
| 1 | EE | P01: I didn't see anything that would be incredibly difficult to understand [about the cessation information]. The only thing I could think of is maybe there [will] be newer vocabulary to somebody. It … depends on the demographics. Personally … I understood everything just fine. | Neutral |
|  | EE | P01: [Finding the cessation information] was easy, it was right there in front of me. Personally, I didn't see any difficulties. What each tab … had inside of it, it’s kind of laid out in layman’s terms right there … I think that it’s really user-friendly. | Positive |
|  | EE | P02: It was easy [to find the cessation information]. | Positive |
|  | EE | P02: No, [the cessation information was not difficult to understand], definitely not. | Positive |
|  | EE | P03: It was easy [to find the cessation information in] that I was just a click away … I wouldn't say there's anything difficult about it and it was easy that you have five different options. You're not just having to scroll through everything … It gets broken down. That also makes it easy. | Positive |
|  | EE | P04: I think that was fairly easy [to find the cessation information]. What was easy about it would be that the button was on the homepage. | Positive |
|  | FC | P04: Me, personally, I probably wouldn't [use the cessation information] because … I have a past with drug abuse, so I've probably read through these things before.^*^ | Negative |
|  |  | P02: I probably would read [the cessation information] if I was super serious about it.^*^ | Neutral |
|  | PE | P01: Maybe [I would use] the staying smoke-free [information], absolutely … With the withdrawal page, maybe [I would use it] if I'm confusing … a side effect of my medication or a reaction to my medication as withdrawal, I would probably go there frequently to double-check … keep myself in reality of, what am I experiencing?^*^ | Positive |
|  | PE | P01: I think [this information] can be incredibly helpful to people who don't know what they're talking about. | Positive |
|  | PE | P02: I just kind of skimmed it, but obviously it is helpful information. I’ve been in other programs for stopping other substances and a lot of its similar stuff that I've read in other literature. | Positive |
|  | PE | P02: Reading it now, there's definitely good information here. It's good to have somebody you could call [using a quitline]. Obviously, take a walk or jog … physical activity is one of the best things. | Positive |
|  | PE | P03: What I found that was helpful [about the cessation information] was … in … the withdrawals and cravings section. It talked about … different options that I wouldn't normally think of. I've always been a cold turkey kinda guy … and then the steps to prepare, it really gives you a list of things you need to do, like talking to your family, et cetera. That was all really helpful … everything about it. Had all the steps that I think you would need. So, overall … it was all helpful, but things that stood out were in step one and step three and four. Just in resources and then really laying down the law of what you need to do to accomplish your task. | Positive |
|  | PE | P04: I would say that [the information is] based on CBT [cognitive behavioral therapy]. So, I think that it would be fairly helpful, and by CBT, I would say Cognitive Behavioral Therapy like the cravings, the withdrawal, the slips … Personally, I think that that is helpful. It's a great way to stop doing something by understanding these things. | Positive |
|  | NA | P04: I don't like the color scheme as well [for the cessation information]. | Negative |
|  | NA | P02: [The layout for the cessation information] was fine, it just looked a little bland … white background, black text … The rest of the app looks good, that page looked a little bland. | Neutral |
|  | NA | P01: I think [the layout of the cessation information is] incredibly organized. | Positive |
|  | NA | P03: Yes, I would use the [cessation information] feature … it was the first thing I clicked on when I was exploring the app.^*^ | Positive |
| 2 | EE | P04: So, it was easy [to set a quit date] because I had already explored the menu previously. If I hadn't explored the menu, it might have been difficult to find it. | Neutral |
|  | EE | P01: I feel like I could accomplish [setting a quit date] but … I couldn't get to the right month … It would be incredibly easy for me to, if it wasn't for that … minor bug … It wasn't even a second thought of where to go … I remember looking at … my quit plan on the menu, and … it’s the second option, it's right there. | Positive |
|  | EE | P02: [Setting a quit date was] easy. | Positive |
|  | EE | P03: Everything was … fairly easy about [setting a quit date]. I wouldn't say there was anything difficult I was able to do it pretty quickly. | Positive |
|  | EE | P03: I wouldn't change anything [about the quit date] layout-wise … I was able to easily get to where I needed to be … so I would not change anything. | Positive |
|  | EE | P04: When you open up the side menu, it's fairly simple [to find] because it says my quit date. I guess if you were trying to set a date, that's what you would click on … It's pretty simple. I think anyone would assume that your quit date is how you would set it because of the arrow here … It's it fairly … cut and dry. | Positive |
|  | FC | P04: Personally, no [I wouldn’t use the quit date feature] … I'm more of a mental planner, as opposed to a visual planner. So, I don't think that I would actually utilize this feature, like go back and see what date I said.^*^ | Negative |
|  | FC | P02: Yeah, [I would use the quit date feature] for sure if I was going to dedicate myself to this app. Of course, I would … put it in there.^*^ | Positive |
|  | PE | P03: I think [setting a quit date is] one of the main features. So, yes, I would use it because it's got … a goal. You need a goal, or mission, to finish what you gotta do. So, yes, I would use it.^*^ | Positive |
|  | PE | P01: Absolutely [I would use the quit date feature]. I think it's important to set goals like that.^*^ | Positive |
|  | NA | P04: Again, I don't like the color scheme [for the quit date]. That's really my biggest complaint at this point. | Negative |
|  | NA | P01: It's a pretty standard layout [for the quit date]. There's not exactly much to say besides … that’s the standard really. | Neutral |
|  | NA | P02: There's nothing negative I can say about [the layout for setting a quit date], it seems fine to me. | Neutral |
|  | NA | P04: I like this layout [showing the quit date] better as opposed to the … layout that we just saw [for information on quitting]. | Positive |
| 3 | EE | P01: [The task was] fairly difficult, especially since … I didn't know … that there was a quitline to begin with, so it’s unexpected information. | Negative |
|  | EE | P02: [Finding the quitline was] difficult. | Negative |
|  | EE | P03: Everything's kind of accessed from the tabs at the … main menu there … I wasn't able to find any resource or hotlink to find [the quitline] so I think it must be in a menu within a menu or something? … Just exploring the app, I was unable to find it either. | Negative |
|  | EE | P04: I thought it would be easy because I thought it would be … under the about application [page] where I would find … a contact number for the quitline. It was difficult because I didn't find it … It wasn't clear how to get to the quitline and what the quitline even was. I assume that that's a phone number. | Negative |
|  | FC | P02: If [the quitline] did cost extra money, I don't know if people would actually utilize it, but … I'm sure there's ways to do it with ads. | Neutral |
|  | FC | P03: I don't know if [I would use the quitline if it] had a cost associated to it. It might rack up if you're going to use it often, but maybe I would use it once just to see what it was all about. I’d definitely use it at least once to … get the full resources you need to help me quit.^*^ | Positive |
|  | PE | P01: [The quitline is] something I've never tried before. Maybe that could be … the missing key that could unlock me from these nicotine chains. | Neutral |
|  | PE | P02: For me personally, [the quitline would] probably not [be helpful] but for a lot of people they do enjoy therapy and talking stuff out, and I think it would be extremely useful for a lot of people if they're seriously using this app. | Neutral |
|  | PE | P01: If it's somebody that's really trying to quit and they kind of took [quitting] to heart, [the quitline] could be something that could really get somebody … out of that mindset of, well … already slipped once today, I might as well slip the rest of the day. I feel like it could prevent that line of thinking. | Positive |
|  | PE | P01: Yeah, [I would use the quitline] … It’s important to … have a support group, and if I can’t get a hold of a friend, that would definitely be a second option.^*^ | Positive |
|  | PE | P04: I probably would [use the quitline], yes … because … if I'm craving, I've learned that it helps to talk to someone about it, like an actual human being. As long as it wasn't automated, I would use it. I would try to use it.^*^ | Positive |
|  | NA | P01: [Calling a quitline], it’s something I would give a good try. It's nothing I ever considered before, but … first time for everything.^*^ | Positive |
| 4 | EE | P01: [The cravings tracking feature] was pretty much right there in front of my face. To be honest, I like that convenience. I know I … went to the wrong page for some reason. I wasn't thinking straight, but that was pretty easy. | Positive |
|  | EE | P01: [The layout for the cravings tracking is] pretty convenient. | Positive |
|  | EE | P02: [Recording my craving was] easy. | Positive |
|  | EE | P03: [What] was easy would be that [the cravings tracking] was the first one of the buttons on the main page. You don't have a lot of options. You just click and click and click and then you’re at the menu I’m at presently. I wouldn't say there's anything difficult about it. | Positive |
|  | EE | P04: [The cravings tracking] was right there on the home screen … It was pretty straightforward when you click it. Gives you this one out of ten. I don't really see anything that was difficult about it. I think it was fairly, fairly simple. | Positive |
|  | FC | P02: If I was taking the app seriously, I would absolutely record the craving.^*^ | Neutral |
|  | PE | P01: Yes, I would [use the cravings tracking feature] … Again, it's something I've never done before, so, yeah, why not go ahead [and] try it, at least for a little bit. Could work.^*^ | Positive |
|  | PE | P01: [The cravings tracking is] pretty useful … I think it would be very informative for people that are looking back on their progress … especially since that could be a good motivation for some … For me, at least, it would give me that sense of accomplishment … I'm actually accomplishing a goal and I have something to show that I am. | Positive |
|  | PE | P01: Yes, [the cravings listed were useful] … at least the few that I skimmed through. | Positive |
|  | PE | P02: [I would use the craving tracking] ‘cause I’m trying to quit smoking.^*^ | Positive |
|  | PE | P03: I would [use the cravings tracking] because … if you can pinpoint your triggers to it, you can pinpoint how to solve your triggers if that makes sense.^*^ | Positive |
|  | PE | P04: Probably [I would use the cravings tracking], yes … Just to remind myself that I'm craving right now and to focus on it, and that you can get through the craving … This kinda takes your mind off of the craving itself by filling out this information.^*^ | Positive |
|  | PE | P04: I think [the craving triggers listed] were [useful], yes. | Positive |
|  | NA | P04: I don't like the color scheme again [for the cravings tracking]. | Negative |
|  | NA | P02: [The layout for the cravings tracking] functions fine. I do notice the app looks good … although all the pages, they have a lot of different colors. It's not … very consistent. | Neutral |
|  | NA | P04: [The layout for the cravings tracking is] okay, maybe I’ll give this like six out of ten … like this continue button, I think it's kind of … intrusive. It could be continue here and close here [at the bottom of the page], but that would be … after I had added the other question so that would fill up the screen more. Then at the bottom, you would have continue or close. | Neutral |
|  | NA | P03: With the [cravings] scales, it made sense and then you have the drop-down and then it leads to somewhere that makes a lot of sense … Layout-wise, I would say that … it makes perfect sense to me … And then you can just close out of there. I wouldn't change much about that, either. | Positive |
| 5 | EE | P03: [What was] difficult [about finding information on distractions] was, if that's what you're looking for specifically you kinda gotta look within a menu within a menu. | Negative |
|  | EE | P01: [Finding information on distractions] took me a second to think about, but it was right there … I saw that cravings was one of the options in the information guide. So, once I kind of put that together, I scroll down, and I see [it] right here … Once you think cravings, you’re probably going to think of that tab if you use this app frequently. | Positive |
|  | EE | P01: Not in the little bit I skimmed through, no [it’s not difficult to understand]. | Positive |
|  | EE | P02: Oh, [finding information on distractions] was simple. | Positive |
|  | EE | P03: What was easy [about the distractions information] was that you only have two clicks, you just got to click … my mood and then you click an emotion and you’re there. | Positive |
|  | EE | P04: [Finding distraction information] was fairly easy … I didn't have any difficulty finding that. | Positive |
|  | EE | P04: No, I don't think so. I think [the distraction information is] pretty, pretty straightforward. | Positive |
|  | FC | P02: Possibly [I would use the distraction information] if I was dedicated to this app … Like I said, I’d have to be like … all in.^*^ | Neutral |
|  | PE | P03: Maybe if you have read it once you've read it twice … you only need to read it once to know the typical distractions. So, it might not be something very informative. | Negative |
|  | PE | P01: Yes [I would use this feature] … Distracting yourself from cravings is definitely important, and whenever you're fiending for a cigarette … whenever you feel like you absolutely need one. It's incredibly hard to … think … hey, maybe I should go for a walk … and clear my head … I think it mentioned a little bit earlier … 5-10 minutes is the typical craving time, which sounds new. [I] never knew that before, but … having that kind of information on hand could … make dealing with it easier and help keep you focused on your goal.^*^ | Positive |
|  | PE | P01: I’d say [the distraction information is] incredibly helpful. I mean it just taught me something new. | Positive |
|  | PE | P02: [The distraction information is] helpful … The select activity one is one of the best ones for me at least. Smoke-free zone, that's easy these days because everyone's outside … Smoking savings, that's a huge one. Save a s**tload of money, do something else, figuring I’d stop immediately to do something else. Great thing, perhaps. I mean, it's nothing … I haven't heard before, but it's good information. | Positive |
|  | PE | P04: Yes [I would use the distraction information]. Now that I know the [quitline] number is there, yes … because I think it's important when you are craving to distract yourself. So, I would say I would use this feature because I think it's helpful.^*^ | Positive |
|  | PE | P04: That [distraction information is] very helpful … I would give it like eight out of ten, helpfulness. I think it's pretty helpful. | Positive |
|  | NA | P02: [The distraction information is] all black and white … it’s not too visually appealing. | Negative |
|  | NA | P01: [The layout for the distraction information] just looks like a normal Word document. It doesn’t really jump out at you … but it lays out everything pretty black and white … I’m pretty neutral on it just because it's pretty standard with … the how-to apps or … pretty much apps in general. | Neutral |
|  | NA | P03: [The distraction info layout] looks good, it's green … I don't get the color scheme of the whole app … I don't know if there's reasoning behind the color scheme of the whole app, but the green looked nice here. | Neutral |
|  | NA | P03: I’d probably use [the distraction information] once … just to see what it’s all about. But, if it's just kind of … go for a walk, et cetera, et cetera … I’d use it once.^*^ | Neutral |
| 6 | EE | P03: [What was] difficult was that … you should be able to click the my reasons for quitting [button], the image perhaps just to be able to quickly get to there from the main page … Otherwise, have it in the drop-down menu, easily able to find it. | Negative |
|  | EE | P04: It was difficult [to add a personal reason] because I couldn't click the link, or when I clicked it, it didn't do anything. | Negative |
|  | EE | P02: [The difficulty for adding a personal reason was] moderate. If the button worked, it would’ve been easy though. | Neutral |
|  | EE | P01: I can’t think of any reason why [submitting a personal reason] would be difficult. I like that there's … room for pictures right there and I'm assuming … it'll probably bring you to … something that you wrote or maybe … it will replace my reason for quitting with your actual reason … Maybe it's just a picture gallery of stuff that's really important to you. | Positive |
|  | EE | P03: [What was] easy about [adding a personal reason] was there's another option. I guess you could just record it elsewhere. | Positive |
|  | EE | P04: It was easy because it said, my reason for quitting [on the landing page]. | Positive |
|  | PE | P01: Oh, a hundred percent [I would use the personal reason feature]. Everyone needs the reasons otherwise you’re never gonna accomplish anything.^*^ | Positive |
|  | PE | P04: I think I would [use the personal reason feature], yes … to remind myself of the reason that I chose to stop.^*^ | Positive |
|  | NA | P01: [The personal reason for quitting is] right there [on the landing page], bold, front, and center, something I can really appreciate. | Positive |
|  | NA | P02: Yeah, definitely [I would use the personal reason feature], especially a list of a couple bullet point reasons.^*^ | Positive |
|  | NA | P02: [The layout for the personal reason] works, it functions, it's not bad. I would grade it a … eight out of ten. | Positive |
|  | NA | P03: I might initially set [the personal reason] up. I’d put a photo of my family … that's the reason you want to quit. So, yes, I would use it.^*^ | Positive |
|  | NA | P03: The layout [for the personal reason], it’s again right when you open the homepage … I like it. I like that it’s got kind of a little paperclip, that adds a little personal touch, I guess … Overall … it’s cute and it makes you do what you need it to do. Alright, it shows you your message, so overall it looks good. | Positive |
|  | NA | P04: I like that [the personal reason is] on the home screen … I suppose this picture would be what you uploaded … I think it's pretty cool, because I guess when you put in your reason, it's going to show up here with your picture. So, I like this feature. | Positive |

| 7 | EE | P01: My phone is being weird with [the wireframe] as well. It's treating [the app] as if it's a picture now [and trying to download it]. It’s not treating it like it's your [app]. | Negative |
| --- | --- | --- | --- |
|  | EE | P03: I don't know if I would [use the personalized support] … I might use it for locations that I don't frequent often, but if I'm at home and it's gonna remind me I'm at home every time, it might be a little cumbersome. So, yeah, I'd use it.^*^ | Neutral |
|  | EE | P01: [The personalized support feature was] pretty straightforward. It was one of the top options. I feel like that’ll catch a lot of people's eyes since … that's not something a lot of people think of. | Positive |
|  | EE | P01: [The personalized support feature was] pretty easy to understand. | Positive |
|  | EE | P02: [Requesting personalized support was] easy. | Positive |
|  | EE | P02: Nope, [the steps to set up the personalized support] were simple. You just click through [the] menu and click the button. | Positive |
|  | EE | P03: [Setting up the personalized support was] easy, I kinda knew the layout. So, I was able to go there, and I think I can just go to my locations. So, there's two ways to get there easily … It's just easy to access … with a lot of options to reach where you needed to be. So, it was easy. There was nothing really difficult about it. | Positive |
|  | EE | P03: [The instructions for the personalized support] were easy to follow … because there was only two, you just have to type the location and it’s there. | Positive |
|  | EE | P04: I think that was pretty straightforward [to request personalized support]. That was easy. I'm finding it hard to tell you what's difficult about each of the tasks. | Positive |
|  | EE | P04: I think [the steps for the personalized support] were easy … because it was like right in front of you. You click the menu, and I would assume that my locations would mean that when you get to this location, this app will do something. | Positive |
|  | FC | P04: No, I wouldn't [use the personalized support] … Just my personal paranoia, I suppose I don't like the idea that the phone knows where I'm at, even though I know it does. I can have a little control by saying, hey, I'm going to turn my location off … I don't keep my location on, so that's why I wouldn't use it.^*^ | Negative |
|  | FC | P04: Me, personally, I wouldn't include [the personalized support feature] … [because of] the privacy reasons. | Negative |
|  | FC | P02: Possibly [I would use the personalized support], I don't use a lot of notifications but if I was all in then I may, depending on how often they send them.^*^ | Neutral |
|  | PE | P01: Oh, absolutely [I would use the personalized support feature] … because … at least part of my issue is the ritual. So, every time I go to the grocery store, I tend to … have a cigarette on the way or in the parking lot after I'm done, or both … Being able to recognize that and … being able to disrupt that ritual by reminding me what it is I'm supposed to be doing I think is … pretty useful.^*^ | Positive |
|  | PE | P01: [The personalized support is] pretty useful and I think it's organized in a pretty good manner, you know. | Positive |
|  | NA | P02: Nothing wrong with [the layout for the personalized support]. It looks like a typical notification thing. The font seems pretty big. | Neutral |
|  | NA | P01: I feel like that's such an excellent idea [to provide personalized support]. I honestly do, and I'm honestly impressed. | Positive |
|  | NA | P03: This part [with the personalized support] is well built … It's just a map. I can hold. I can type. So, I think that the layout’s good. It's got all the options you need … to get the job done … It accomplishes what it sets out to do. | Positive |
|  | NA | P04: I think [the personalized support layout is] okay. I think it’s about as good as it could look. | Positive |

| 8 | EE | P04: I would say maybe the “you saved yourself 2450 minutes” [could be difficult to understand] because that could mean two different things in my opinion. | Negative |
| --- | --- | --- | --- |
|  | EE | P04: The easiest part [to find the tracking page] was … this little [graph] image or emoji, it's kinda … universal for statistics, and it was also easy because it was included on the home screen, and you just click it and it kind of slides out. So, fairly easy … The only thing that would be difficult, I guess, is … if you [weren’t] familiar with this. | Positive |
|  | EE | P02: That was very easy to find [the tracking page]. | Positive |
|  | EE | P02: It was easy to understand [the tracking information]. | Positive |
|  | EE | P03: [Using the tracking feature is] easy … it’s right at the top there. I think you program in all your stuff, but … it was easy ‘cause it's a click away, it’s right on the homepage. | Positive |
|  | EE | P03: Nothing seemed difficult to understand [about the tracking feature] … Overall … really easy to understand and not difficult at all. | Positive |
|  | PE | P04: The easiest part [to find the tracking page] was … this little [graph] image or emoji, it's kinda … universal for statistics, and it was also easy because it was included on the home screen, and you just click it and it kind of slides out. So, fairly easy … The only thing that would be difficult, I guess, is … if you [weren’t] familiar with this. | Positive |
|  | PE | P02: Oh, [the tracking feature is] useful. | Positive |
|  | PE | P03: I’d use [the tracking feature] often because … you can really track in real time … how well you’re doing.^*^ | Positive |
|  | PE | P03: [The tracking feature is] helpful ‘cause … it gives you the real numbers instead of just resources. | Positive |
|  | PE | P04: I think … [the tracking information] that's good, that's helpful … I like that. It includes the dollar amount … and I guess the minutes would be … how much longer you're going to live? … If that is what it is, I like that aspect and I also like that it counts … cigarettes [not smoked] and your days. | Positive |
|  | NA | P02: [The layout for the tracking information] was good. | Positive |
|  | NA | P02: Yeah, absolutely [I would use the tracking feature]. It'd be one of the main features.^*^ | Positive |
|  | NA | P04: Yes, I would [use the tracking feature] … I like the ‘you saved and the dollar amount.’ | Positive |
| General | EE | P01: I'm kind of actually curious, what is my location [on the side menu]? My location … is that kind of … keeping track where I might have the most cravings … that kind of confuses me right there. | Negative |
|  | EE | P02: One confusing thing was that picture thing [with your personal reason for quitting], but I'm sure I'm just … not thinking about it, frankly. | Negative |
|  | EE | P01: Location, time of day, history. That seems really convenient … yeah, it's easy to use, I think. At least that’s the way [the side menu] looks. | Positive |
|  | EE | P01: [The tracking mood and craving buttons are] in convenient spots for my thumbs. They're not … too glaringly bright or anything, they’re pretty soft colors. I like the fact that … manage my mood is green, since green’s usually associated with … positivity or go. | Positive |
|  | EE | P03: If this is an app, it looks good … Right at the start there, you’ve got the I was smoke-free today, I slipped [buttons]. Kind of like a directive right there, easy. | Positive |
|  | EE | P03: [The cessation information is] not a lot to read, which is nice ‘cause I don't know if everyone wants to read. | Positive |
|  | FC | P04: My initial thought was, I looked at the word quitism. I’d never heard that word. It could be a little bit more graphically appealing if that's the right word. | Negative |
|  | FC | P04: I would not use [the app] personally, and the reason why [is that] I don't use my phone a lot to begin with … see that's the main reason. But it would be the personal paranoia of the location.^*^ | Negative |
|  | FC | P04: Personally, I don't like the idea of the app following my location, so I would probably not turn the location on. That's probably just my personal paranoia, I'm sure some people would like that feature.^*^ | Negative |
|  | FC | P01: I just [have] never seen anything like [the tracking feature] before. I never even considered writing it down in a journal or calendar or anything. So that's just … a new idea to me. | Neutral |
|  | FC | P04: To me, it looks like a pretty standard menu I've seen on many other apps. | Neutral |
|  | FC | P01: I don’t know if exactly right now is the best or worst time to quit smoking … with everyone being on quarantine … You got the stress, but you also have all the stores closed. It’s a coin toss over here for me, but if the world goes back to normal, or at least relatively close to, in the next few months, it would be a high likelihood that I would use [the app to quit smoking].^*^ | Positive |
|  | FC | P02: Yes, [I would use this app] because I am interested in quitting smoking. I've tried before and I never actually tried to use an app with it before, so I would consider giving it a shot.^*^ | Positive |
|  | PE | P01: Learn to quit at the bottom is … pretty standard. You see that kind of everywhere. Nothing really stands out there. | Neutral |
|  | PE | P01: So far, it's stuff I've heard before, but not everyone has looked into quitting, so I feel like this [information] could be very useful to those who haven't had any … so far. | Neutral |
|  | PE | P01: [The information is] relatively speaking, stuff I've seen before but … I've also looked into … quitting smoking before. But I know a lot of people … for example, my mother, she's never looked into it a day in her life. She … [keeps] saying how she wants to [quit] but she doesn't know exactly what that entails, so I feel like this would be really incredible information for people like her that are kinda all talk but no information. | Neutral |
|  | PE | P01: That quitism … seems like that could be … good motivation … that looks like that could be pretty useful … [The] my reason for quitting, I feel like that could be a great source of motivation … That's really the only thing that pops out on this [landing] page. | Positive |
|  | PE | P01: Maybe you tend to have the most cravings on your way to the grocery store, so I could see [the personalized support] maybe being used for something like that. Yeah, kinda keeping track of where … The biggest problem with smoking isn't necessarily the addiction, it's the ritual … I could see that as being a useful tool to … figure out where your ritual spots are and how to change that. | Positive |
|  | PE | P01: I was smoke-free today [and] I slipped is a great way to go ahead and keep track of … your daily progress. I like how my reason for quitting is right there … front and center, that is pretty nice. | Positive |
|  | PE | P01: Track my craving, track my mood, I mean they look useful. | Positive |
|  | PE | P01: I think that [tracking] could be incredibly useful. | Positive |
|  | PE | P01: I think that [tracking is] actually a pretty good idea, the track my location and the craving tab in general seems to be very interesting. I feel like that could be incredibly useful, especially with … figuring out where your ritual spots are. I think that could be proven to be incredibly useful. | Positive |
|  | PE | P01: [The suggestion] of encouraging people to develop a support group, I feel like that would be incredibly beneficial for a lot of people. | Positive |
|  | PE | P01: In all honesty, that's actually not a bad layout at all. The tracking my cravings and … manage my mood … a lot of people don’t look at the mood aspect. That’s what my biggest issue is. I’m normally a level-headed guy, but if I don't have nicotine in me … I’m not angry, but I'm more frustrated with everything … the manage my mood has really spoken out to me. | Positive |
|  | PE | P01: I also like [that] it’ll let you keep track of … [being] smoke-free today [or that] I slipped. I never actually tried writing it down … if I slipped today or not, so I think that's incredibly useful. | Positive |
|  | PE | P02: That's good, it shows you how much money you saved. | Positive |
|  | PE | P02: That’s cool, you could pick your own triggers and stuff like that. Might be useful if you can add your own too. | Positive |
|  | PE | P02: You can share [your quit date] with your friends. That's very useful. | Positive |
|  | PE | P02: I think it seems like a very useful app. | Positive |
|  | PE | P02: Move around while you talk, or change of scene really help, go outside … that's good advice [during a craving]. | Positive |
|  | PE | P03: Seems like there's a lot of good information you wouldn't be able to find unless you really seek out that specific information. So, it's a nice to kind of have it on the app. | Positive |
|  | PE | P03: I would [use this app] ‘cause I think it has some information that I can't find on other apps … It sets out to do the task it needs to do, so yes, I would use it.^*^ | Positive |
|  | PE | P03: I’d hit learn to quit and then I'd read through all these [tips] … Good information because I didn't know about these pills. That might be something I'd want to look into. | Positive |
|  | PE | P03: I don't know if this [advice] would be different per mood you pick, or if it’d always be the same. Call a friend option’s something I wouldn't expect to see but I guess could be very useful. | Positive |
|  | PE | P03: View notes and photos [after selecting a mood], kinda just distractors. That's nice and then you could change your reason for quitting, I guess you wouldn't change that often, but I like that you can put maybe a photo of your family or something, or you want to save money for a vehicle, or whatever you're saving money for. | Positive |
|  | PE | P03: The manage my mood [feature] was a lot better than I thought. I liked that you could just look at other things on your phone or just really distract yourself, call a friend. That's not what I would have expected to see and I kinda liked it. | Positive |
|  | PE | P03: I also liked the learn to quit [feature]. It had a lot of resourceful information. | Positive |
|  | PE | P04: I think [the app] could be helpful for someone … trying to quit smoking. | Positive |
|  | NA | P01: I see there's a little bit of a [graphical] overlap on the app … I don't know if that's … because I'm on this website version or if that's a programming error, but that could … have some people question [its] credibility. | Negative |
|  | NA | P04: I don't really like the colors personally. | Negative |
|  | NA | P04: I personally don't like the color schemes. That’s the only complaint I have so far. | Negative |
|  | NA | P04: I don't like the color scheme [of the app]. | Negative |
|  | NA | P03: The quitism … I don't think I’d use that as much, but some people might like that.^*^ | Neutral |
|  | NA | P01: I think it's very soft. I really do … It’s not like most quit smoking ads I see that [have] very … bright colors … Those brighter kinds of eye-catching things are a little bit more aggressive, and this seems to be a little bit more soothing, a little bit more … on my side. | Positive |
|  | NA | P01: I’d be excited to download [the app] and try it. At least give it a good shot.^*^ | Positive |
|  | NA | P01: I think you guys are doing incredible work. I see a lot of potential in this app, and I am honestly really hopeful that you guys continue to work on it, buffer it out, and make the best possible product because I would definitely be buying it or downloading it.^*^ | Positive |
|  | NA | P02: I like the layout. The colors seem to look fine to me. | Positive |
|  | NA | P03: I like that it's black. Not a lot of apps have this kind of color scheme. | Positive |
|  | NA | P03: [The red for] I slipped is a good color. | Positive |
|  | NA | P04: [The app] looks pretty simple. | Positive |

Participant identification number appears before each quote for attribution.
^*^Indicates quote references intent/willingness to use.
EE= Effort expectancy, FC= Facilitating conditions, HM= Hedonic motivation, PE= performance expectancy, SI= Social influence, NA= Not applicable.

**Supplementary Table 5.** **Illustrative quotes of smokers’ suggestions for improving Quit Journey and QuitGuide.**

| **App** | **Quotations** |
| --- | --- |
| Quit Journey | P08: This part is a little confusing … but when it says help me next time I'm here [for the personalized support], I don't know if they mean like help me next time I’m in this section of the app or if it means … like help me, like give me a notification … when I'm … feeling a certain way … explain that a little better or something. |
|  | P08: I feel like there would have been … a little more … to the [mood tracking] section going a little more like in-depth on … the craving and … how to stop it or … what kind of like notification to give you when you're … feeling that way or … how you can deal with it. |
|  | P08: I think maybe [adding] … like a contact section or something where … you can be … more like hands-on with … another person … I know the tips and … the advice that's nice because it's … anonymous but it would also be nice to … kind of … go back and forth with … some kind of … smoking counselor or somebody who's … running this app who has … experience in … dealing with this stuff or … maybe somebody that's … handling the app's features and stuff. |
|  | P08: I feel like maybe if they had … a video or something that explained a little more on … how it worked … for people who've never used a smokerlyzer before, like to show … all the different ways that you can use it and … all the like different ways to … work it with your app and … kind of work with it in that way. |
|  | P08: I think besides maybe adding a little more of an explanation on how to use the smokerlyzer, I think it's all pretty simple and pretty straightforward. |
|  | P08: I just think, just to be … extra, a video would be nice to like explain [the carbon monoxide monitoring] more. |
|  | P08: I think if they're … trying to make this section … like a messaging section or somewhere where … people can … get messages that … make them feel more comfortable or something like that then I think maybe it should have its own section or not be … with the challenges and the inspirations and all that type of stuff because I feel like that stuff isn't really similar … The inspirations are kind of similar, but I think the challenges are a little too much. I feel like the challenges maybe should have their own section … I feel like that section should be on the homepage and for messaging. I feel like tips and inspirations should be paired together, because those … seemed like they kind of have … a similar feel to them with like how it makes you feel. |
|  | P08: To be honest, I think I would [use the message library] if it was a little more straightforward … I think it would be nice if it was set up in a way like … social media or … Instagram or Twitter, for example, where you can just … scroll down. |
|  | P08: Making a feature where you can go back and forth throughout the different tips and inspirations [would be useful]. |
|  | P08: I don't know if … this app has … a contact section or something where you can … talk to … a physical person or … maybe something like that … Like a contact page would be kind of nice or something similar to that … Maybe like the customer service … workers or somebody who’s … working for this app kind of engaged with … their customers or whoever’s engaging in this app. |
|  | P06: Maybe if you see how there's … the bottom part of the [landing page] that's [blank], that's like the white … I think maybe if you did the money saved there and made it a little bigger, I think that that would be kind of cool because … the money that [you’ve] saved is kind of … a big motivator for quitting smoking. So, I think … if that was moved to the bottom part and made … bigger, and maybe … a different color. |
|  | P06: Maybe you could move the whole thing up a little bit and make the I was smoke-free today and I slipped … maybe [make] that a little bigger too because I feel like the top [of the landing page] looks kind of like empty. |
|  | P06: I think, actually, right at the top [of the landing page], that would be cool if you were to have the little messages right at the very top. |
|  | P06: I think instead of that clock [icon for I slipped today], I think it might be kind of cool if you had that little … no smoking logo … like the typical … cigarette with like … the red X … I just think that white [space] is … really empty … I just feel like there should be something there. |
|  | P06: I think graphs would make it look nice … showing … the progress that I've made. So, it's like … in different ways. I think a line graph would be cool … or like regular bar graphs so seeing … how many cigarettes smoked one day. If I smoke two … just seeing the progress of the days and … you could look at it through a day and see when you smoked a cigarette, how many you smoked at certain times, or … over the course of a week. Then same thing with the money saved, I think that should be something clicked on and maybe that could be like, you click on that, and it shows you how much money you saved and then it could say something like, after quitting smoking for one day you can save $10, after a week you can save $70 … just have little milestones like that … oh it would be cool if you said … I haven't smoked in a year and say I saved, I don't know the math and I'm just gonna say $700. You could write things like, you could buy … some $700 item or some $50 item. Like, you could have bought this with the money that you saved. |
|  | P06: You could ask people what their motivators are. So, if they like technology or like crafting, like for me, I like crafting stuff … and then … you could input it … Say I wanted to travel, I could write my main goal would be saving … X amount of dollars to do this, to go to a different town or something … It could say, you are this close to this goal. You are this close to going to Boston. You are this close to buying … some item that you choose personally … If you wrote what you wanted and then set the price of it … it'd be like two more days until you can buy a printer … or a videogame. |
|  | P06: So, obviously, the reward would be for not smoking for longer, but if people could set their reward, so instead of like the $20 one, set a $5 one for being smoke-free for a week … and then the $20 one could be smoke-free for a month … so people can choose their own rewards because … where I'm at right now that lower one would be more of an incentive because I am still actively smoking, as opposed to like a month seems so unachievable, like that seems so big and far away. |
|  | P06: When I click record [my mood] … it just kinda … went to the main screen. If it said … maybe a little thing that says recorded … just something like that because … I don't know if it did record or if it's just kicking me out. |
|  | P06: I think maybe having … more options for mood, because … people aren't black and white … So, you could do like a happy, sad graph like this, or you could do maybe, like an anxious or stressed one, and you can move it over to how anxious or how stressed you are just to have … a couple different feelings and then have those … maybe four … Instead of like this happy or sad graph like that, have it just be a sad one. I can scroll up from 1 to 10, happy … 1 to 10, anxious, 1 to 10 … stressed. So, that way it goes a little more than just happy or sad, because I feel like … it doesn't feel like it goes in depth enough, I guess. |
|  | P06: Maybe just put some color to [the mood tracking page]. |
|  | P06: I think maybe if you had an option that says … your moods, and you could click on that and see what moods you've had and what caused you to slip over the past … X amount of days or day. So, like say in the morning I smoke a cigarette because I was angry and then [in the] afternoon I smoked a cigarette. I was feeling neutral, for no reason I just smoked. I think it would be kind of interesting to see how [over] the course of the day … how my emotions correlated to smoking throughout a day, or a week, or a month. So, you had, like, a week, it could say, you are happy, X amount of times, you are anxious X amount of times. |
|  | P06: Maybe instead of just having [all the buttons] be boxes, maybe more like circles or … having it at the top of the screen and have the mood at the bottom of it and have them all listed out. So, happy, sad, anxious. So, have that all listed out and then have these buttons, could be like a little smaller, so you could … see your mood more … and then maybe still what caused you to slip, you could have a drop-down menu. So, yeah, what caused you to slip, and it had … a list of typical triggers. So, morning coffee, being upset, being stressed … just typical drop-down triggers, but then you could also have other, and then you could put that in, so that way … if it was a drop-down menu, that would be another way to track it instead of just … I wrote that I was angry for three days you could monitor, you could track that. But, if I actually had to type it in … as an app, it wouldn't be able to … recognize that I did the same thing multiple times, for the same reason. |
|  | P06: It might be kind of cool if there was a map … that showed your location and showed where you were … So, I can manually set up … if I'm in this location at this time, to do it … I think that would be kind of … useful if I can manually set it, instead of just, hey, I'm here, stop me here. So, that means, like, say, I'm at home. I could set it to send me reminders … when I'm at … home about my work instead of having to physically be at work and already have that kind of craving situation. |
|  | P06: This [menu with the] get a tip, take a challenge, play a game [options]. I think that should be on the home menu … Maybe if you moved all of this stuff upward a little bit, and then you could have it maybe in that whitespace [on the landing page], or having it kind of like down maybe… I don't know actually, or maybe you could also have it in the drop-down menu … because … it feels like you have to do very specific things to get to this menu … It feels like it should … be able to … drop-down here. |
|  | P06: It feels like maybe you should have a spot where you can write your things here. So, maybe like a notebook page where you can write your favorite quotes and then you could use that to cycle into those like things that pop up. So, all the tips aren't just from … random people … all the inspiration isn't just from random people, I could … put a couple in, so it would … pop up randomly … when I needed the inspiration. |
|  | P06: Like I said before, I think having [the craving triggers in] a drop-down menu [on the side] … I think everything should be in this drop-down menu … because I feel like it takes a little while to get over that learning curve of the app, but if everything was in the drop-down menu, it would make it easier to use. |
|  | P06: I think [the history feature] should just do the cigarettes more … like a separate section where it says all … the cigarettes that you smoked or didn't smoke … I just think a graph would make it more easier to see your progress as opposed to just … a list. |
|  | Oh, what might be kind of cool [is] seeing other people's goals. So, being like Jennifer S from Utah just went two days without a cigarette … maybe like a little scroll thing at the bottom [of the message library page], saying that? … You know so you could see … what other people have accomplished, and that might feel like, oh well, they did it, I can do it. |
|  | P06: Yeah, I do think [the game] could be more fun. I think it should have different games though, instead of just this one. So … like the typical kind of Candy Crush style game. I think maybe … a style like that, like just having a couple different games, like a bubble pop kinda game. Like just games that people like already. |
|  | P06: I think [the game] should be in this little drop-down menu [on the side]. |
|  | P06: Maybe instead of going right into the game, it [could have] a menu where it had … you could pick like the three games. |
|  | P06: Maybe having different gift cards to choose from, so I could choose a $5 Wal-Mart card or a $20 Amazon card … Obviously, you'd have different things you would need to achieve to get to that point. |
|  | P06: I think a map kind of in the middle screen [for the personalized support] would be a good idea. |
|  | P06: The games, maybe the I slipped craving, inspirational messages or messages, advice, graphs [should be in the side menu]. |
|  | P07: Maybe [add] some color to everything [on the mood tracking page] … it's pretty black and white, outside of the blue buttons, maybe like … a red to green scale from 1 to 7 … I don't understand at all what these two help me next time buttons would be, I would just get rid of them because I don't even know. If there was like an “X,” I could press to get rid of that anytime I went to this page I would “X” out of it because I have no idea. |
|  | P07: I think maybe something … like a live timer would be kind of cool, that might be a cool feature. Instead of just a log [of days smoke free], you have an actual running timer … I think that might look cool. |
|  | P07: If it is what this whole screen is for, it's about … whatever the content in your blood or your breath is, I think it would be important to have some sort of … a question mark icon somewhere that people can click on to get a better understanding of what exactly these numbers mean and … just kind of a breakdown … of what it means, so people know exactly what the number means when they actually do it. |
|  | P07: As long as I'm not getting notifications or being bothered by [the message library], I can just ignore it … So, as long as I have the option to always ignore it, I have no problem with it being in the app, if it helps other people. |
|  | P07: I'm assuming when it's a finished product you would do something like … click on something somewhere and it would bring you like a drop-down menu of your options of what you can choose from to redeem the reward. |
|  | P07: I would rather the app just assume every day that I was smoke-free unless I said something otherwise. |
|  | P07: It's a little confusing just because … when you click the menu here … when I click on smoking details, when I go back, I'd expect to be back in the menu, but instead it takes me all the way back to [the] home [page]. So, there's no difference between pressing home and back. I kind of wish that was different, at least they were two separate things, so I didn't have to continuously go back into the menu to find my last page. |
|  | P07: Okay, yeah, this is what I assumed the awards thing was gonna be, just milestones … outside of basic milestones, I guess, it could be nice to see, but I’d rather just have the numbers that show me exactly how many days or how much money I've spent. |
|  | P07: Can't click on any of these [tracked behaviors on the landing page] … if this were an app, I'd like to be able to click on something like time saved and it’d open up a menu that has a more detailed breakdown, instead of just the minutes, like it should say … X amount of months, or X amount of days, different … logs of something like that. Same with any of these [tracked behaviors] … If I could, I would like to be able to click on this and not only would it tell me how many cigarettes, but then how many packs or … just a more detailed breakdown of each aspect of this app when I click on these, but I guess I can't click them, except I can click I’m craving. |
|  | P07: I'd assume with … the finished product, it would just be like … a list you could scroll through of the different companies that offer gift cards you can redeem, is my guess. So, I’m assuming … the finished app would have a bunch of … logos or just lines of text explaining what each gift card offers. |
|  | P09: The blue [on the landing page], maybe if it was … a little bit contrasty-er. |
|  | P09: Maybe if [the] awards screen didn't have the same symbol as … [the] about Quit Journey [page] … that would … make it maybe more … trustworthy, ‘cause it's kinda like [the] awards screen looks like a notification instead of … what I think it would be, which is like the list of times I've been rewarded for doing something. |
|  | P09: [The] check rewards [icon] is more like a notification … I overanalyze a lot, but that to me, I feel like they should have different symbols, each [option on the side menu]. That kind of makes it confusing. |
|  | P09: Identify smoking triggers and plan how to handle cravings [in the quitting tutorial] I wish maybe plan [how to handle cravings] had … a symbol or something ‘cause it just kinda looks different. It doesn't look finished right there, looks kinda like maybe this is the apps that end up malfunctioning after a while because … some things are missing. |
|  | P09: I feel like, yeah, the [personalized] support would be in I slipped today … and also, for some reason, also, I'm craving, because I slipped today is kinda like, oh my God, like I'm confessing, now I've slipped and I need support anyway because I didn't just keep that to myself, I'm now saying it to the app and recording it … Then also, I'm craving, because I would kinda want the support to help me not smoke. That's the main thing. |
|  | P09: [The] my current mood … scale is nice, but I think some colors would help for the smiley faces. |
|  | P09: I would need to know, like … with an asterisk … what is [PPM] and … what my peak should be, or what it shouldn't be? And what is the average human beings’? And why is mine so high? Why is mine so low? I don’t know. |
|  | P09: Hopefully when you do scroll sideways [on the messages], it stops and doesn't like continue to slide too far, and according to the ones that you want to click. |
|  | P09: [The app] should also … record how old we are because … for some reason it looks adult-friendly … If maybe I was … 15 and I'm … seeking to quit before my parents find out, the colors and everything would make me feel like this isn't really adapted for me. So, maybe … it would know … how old you are. |
|  | P10: I think I was smoke-free today should be green, not blue … because green is positive … if you were going to do a blue, I’m smoke-free today, you should make the color theme blue or [have] it … where people can change the color themselves or the things themselves. |
|  | P10: There's no help information inside. I’d assume that there would be … a help button somewhere or an order button. Somehow, you'd have to get that. |
|  | P10: One through 7 [for the mood tracking], That’s an odd number too. You don't ever see 1 through 7, 1 through 10 or 1 through 5 … 1 through 7, I’d change that to 1 through 10. |
|  | P10: Maybe tell … why you're so happy or why you're not so happy … and then maybe you have another area for craving, do you have a craving? Yes or no, and then another spot will open if you have cravings. |
|  | P10: Go through your Apple Watch. It will give you a notification with … whatever it's testing for … so you don't have to open up … the app all the time [to monitoring your carbon monoxide], but maybe once a week or whenever you want to open it. |
|  | P10: I would change if [the carbon monoxide monitoring] was in the app to where you won't have to use it in the app. |
|  | P10: [The smokerlyzer] should be Bluetooth [connected]. |
|  | P10: Giving you the ability to have these [messages] sent to them via text message … Have one of these come to you every day that’d … be alright, or a notification every day, something that boosts their confidence. |
|  | P10: Maybe give them a little bit more options or menu options [in the message library]. I don't know how you would do that … but just more options for the user to go through here … I have no idea, just more … general topics, I guess, like … craving topics or help them in quitting topics. |
|  | P10: Have leaderboards and … all that good stuff to keep people intrigued … it would go more so with the ranking system or the awards system … So, maybe, using that breath test you can earn the awards through a leaderboard. So, one in one hundred people that get onto the leaderboard in your area, or maybe in the United States, or just in general, like globally, you can earn award points, or maybe something along those lines. |
|  | P10: Maybe, just a forum for people to talk to. Like, communicate to about helping with quitting, just more so than just advice. Being able to talk to other people … well the people that are on the app, trying to quit. |
|  | P10: Something I would add [to the game] is a lot more different things to add in to preoccupy … your mind, so you'd have a bunch of fruits and only one fruit that you can click. That would have only a healthy option. I would keep the cigarette reference out of it just because you don’t want to be thinking of a cigarette, you want to be thinking [about] playing this game. |
|  | P10: That [game is] kind of self-explanatory, but if you wanna add instructions then go ahead. Just says, touch the oranges. |
|  | P10: I don't see why you guys would keep this [game], being that it should say … healthy choices only, or something along those lines or give them more direction or instruction along the [game], because just touch the oranges, okay. That piques my interest for one, maybe two games. Now, if you have a bunch of stuff, healthy options, better game to play, maybe like a toon-blast where a bunch of stuff comes out, you can do all that stuff because everybody gimmicks off of that … put a toon blast kinda game in here. |
|  | P10: The speed and the options of what you're clicking [in the game should be changed]. So, as you keep going, and you slowly progress, instead of after so long of you clicking something and then it’s just game over. Like, I know … you're having a great time, watch me play this game … I'd probably start a Twitch with just this one right here. |
|  | P10: [I would add] what I'm gonna get paid, like honestly, because it's all about the money. |
|  | P10: I would never change it to where people earned gift cards or anything other than PayPal balances or like actual money because that's what piques people's interest, and if you guys are actually paying out real money, that [gives] people [a] real reason to give you good reviews. |
|  | P10: I don't know if you heard of an app called Sweat Coins, but the way you earn rewards in Sweat Coins is by having your phone in your pocket and walking. That's it. You walk and those steps get turned into currency at the end of the day … With your device, you can do the same concept. If people are using your device, then they get points at the end of the day to build up toward a $20 coupon or a $5 coupon. |

| QuitGuide | P01: I like how you guys have call a friend … That's another thing that a lot of people aren't thinking about, trying to set [up] that support group. So, that right there really jumps out to me, and I feel like that should be a little bit more front and center, because … that's not going to be a lot of people’s first instinct, even though it should be because quitting’s hard and you need a friend sometimes. |
| --- | --- |
|  | P01: With the call a friend button, where that is, I feel like that should be a little bit more front and center. That's the only issue I have with it. |
|  | P01: Is it gonna to send you notifications, like … you've been smoke-free for three days … Is it going to send notifications like that? … I'm very responsive to positive reinforcement. I understand that's not everybody but that's just me personally. |
|  | P01: I would probably put [the quitline] somewhere with the I slipped [options] … make that one of the first options … call a friend or call a counselor, something like that? |
|  | P01: One idea I do have … maybe also recording the time of cravings and kind of have … pop up reminders … you get a pop-up reminder the most often times you get cravings and then whenever you tap the notification it brings you to this [distractions information] page. I feel like that could be incredibly helpful for a lot of people, myself especially. |
|  | P01: Maybe a handful of pictures and … have them change … periodically from a handful of pictures that you put there … Seeing that rotation of pictures … periodically would definitely keep me motivated without a question … If I were to click on my reason for quitting and see … a little paragraph I typed up … I feel like that could be very useful right there, along with the ability to … add text as you go … this was my reason for slipping, but this is my reason for rebounding. |
|  | P01: Having a … reminder … rather than smoking, here call a friend … Here’s the how to curb my cravings page or distract me from my cravings page with the number for that counselor or something like that to kind of … remind me [because] it's second nature … but to have that reminder … don't do that, here's how to not do that. I feel like that could be incredibly useful for at least me. |
|  | P02: You could post [your quit date] to Facebook and stuff like that because encouragement helps, I noticed a lot like people losing weight and stuff. |
|  | P02: I think more of this generation would prefer to … have [cessation information] in like a little video form, even if it's just like a couple. You [get a] couple of voice actors, some animators in there, and it reads out loud for you … Not that I’m against reading, I'm just talking about the current generation that … use apps. |
|  | P02: [The listed cravings are] fairly comprehensive, but I think … it’d be useful for the user itself to add … one that's not on here … Maybe even have like other people that use the app, they usually contribute and like … oh yeah that’s a trigger of mine, it could be added to the list. |
|  | P02: It'd be cool if the craving bar looked like a cigarette itself … just for some aesthetic purposes. |
|  | P02: [The distraction information is] kind of a wall of text, it could be separated more by paragraphs. |
|  | P02: The [distraction information] page … could be presented in a … more modern way. |
|  | P02: Maybe even have … your family members be able to comment on like, oh, I stopped this craving or something … They can leave like a thumbs up or a good job or something … some social feedback. |
|  | P02: I don't know if there's any way to … provide any kind of real incentives … I know there's … to other apps like … fitness ones … to … work out … I don't know if you could work out some kind of incentive to … actually make some money back by not smoking and using the app. |
|  | P02: All the information … maybe present that not all at once … but throughout the app [as a pop up] … like here's another factoid about this part of it. |
|  | P02: It could be easier to find the location [to] connect to the counselor. |
|  | P02: Right now [the app] gives you a piece of advice here [after tracking a craving], but I know it's the same advice as before, so you might want to rotate more of these. |
|  | P03: The learn to quit [option] … looks like just to quit in general, like [a] leave the app button … I might change the words on there. |
|  | P03: The buttons themselves, maybe I was smoke-free today could be green, whereas manage my mood could be blue … It's kind of like a positive reinforcement type of thing. |
|  | P03: [The] learn to quit [button], maybe I would give it a different color besides the gray on black … color scheme. Maybe if you’re choosing darker colors, like a brown or purple. |
|  | P03: I don't know if there's an option to put in a reason for [craving] yourself, maybe there's other reasons people would do it. |
|  | P03: It should be maybe seeking out partnerships with other companies like the Lung Association or … Smoker's Help Line … it just kind of creates more of a community … Say the app’s not working for you that day, maybe if you have another resource to click to, and that's not like a really big thing … I think the apps got a good setup here … but if I was going to add something … maybe a partnership or something with another company. |
|  | P03: [The layout for the cessation information] looks good. You kinda got a blank space underneath that divides the screen in half … maybe put an image there. |
|  | P03: You might also want to just put a calendar [to the quit date feature] that you can just adjust the month, week, date … Like a full-fledged month calendar view. |
|  | P03: I don't think I would add or change anything [about setting a quit date] besides that little calendar thing or being able to click the big [date] numbers there. |
|  | P03: I would put [the quitline] maybe in the [main] menu there, when you initially click it to have it in the drop-down here. |
|  | P03: [The craving triggers were] comprehensive, yes, but I noticed there's not an option to add your own. I would … add an option to add my own, maybe this location, this time of day … just a straight add my own to it would be useful as well. |
|  | P03: Maybe I would include that option to contact the help line as well [as an option after having a craving]. |
|  | P03: If you're looking offhand to find tips and distractions … maybe I would put it in the drop-down menu or something because I feel like that's something that you would be looking for often. |
|  | P03: If it's something that's going to have a lot of distractions for you built within the app, I would have an easier access point to it. |
|  | P03: [The] “what would you like to do?” [text on the mood page] … maybe I'd make it a little more bigger and bolder. You can’t really see that’s there. |
|  | P03: I would just change the way to access [the personal reason], that’s about it. |
|  | P03: Maybe I would set … [a] do not remind me in this area thing, but I don't know if it does that anyways. |
|  | P03: Maybe I’d add an option to adjust the settings for [the tracking feature]. |
|  | P03: If I was to change the word, learn to stop, I don't know … quit works. |
|  | P03: I still don't know what [the my locations option] would be about … So, maybe I would have just kind of a little introduction to what my locations means. |
|  | P03: Maybe have the resources being found in like a drop-down menu … I would add a fifth … option to it … like just another button that says talk to a professional. |
|  | P03: Maybe if there’s not enough options on the list of distractions, I’d add more things. |
|  | P04: I would add like a meditation feature … Like after cravings, maybe number three [in the list of information topics] would be … how to beat the cravings, but it may be included in there … I think I would put six instead of five [topics] and so, where three would be, I would put like overcome cravings … I would not number it. I would use like a bulleted list. That's what I would personally do. |
|  | P04: Maybe your quit date could be on the home screen. |
|  | P04: There could be like a calendar on the home screen that would include the [quit] date as soon as you open the app. |
|  | P04: I like the black [color], but the white text, I would have yellow text and then in the background, how the background is blurred, I would probably put … a person smoking or something … and … looking sad or something like that as … a visual cue. |
|  | P04: I think that it should [be on the] home page … I don’t know how it would fit it, but maybe … like, call the quitline, you’re struggling. So, it would just be right in front of you. |
|  | P04: I would maybe add another question [to the craving tracking feature]. After how strong is your craving, I would include why do you think you are craving before you select your trigger and then you can compare. |
|  | P04: [The quitline] number would have been on the front screen, the home screen, so this wouldn't be here [with the other distraction information] … I, personally, like bulleted [lists], so … like the first sentence, would just be like the list, you could click the bullet, then the next screen would be the paragraph. |
|  | P04: [The distraction information is] kinda crammed together, like it can be more spread out, I suppose. |
|  | P04: [I would change] the colors. I don't like the block [of grey] necessarily [for the personal reason]. |
|  | P04: Make [the personal reason layout] look like it's floating … If [the landing page] wasn't black, so … like the background didn't absorb it, it can be … a yellow font, and it should be somewhat floating here without … the black look behind it and then the picture just floating as well, I guess that's the correct term. No background there, I’ll say that. |
|  | P04: [I would change] the color scheme [on the tracking page]. |
|  | P04: I like the bulleted type layout. See … how these [where it says your money and time saved] almost look like buttons … why don't [the days smoke-free and cigarettes not smoked] look like buttons? … So, maybe it should all be the same. |
|  | P04: I would personally probably add more options here [for the notification controls]. Like … if the user wanted just a picture to popup, which me personally I wouldn't, but that would be cool. |
|  | P04: [The cessation information] could be wider on the screen … if it was a bulleted list, it could go into more detail I suppose … When we click on this, we have to scroll through the whole thing, but if we had like a bulleted list and then subcategories you could click that link and go straight to that part of the section. |

Participant identification appears before each quote for attribution.
